# Ventilation of the abyss in the Atlantic sector of the Southern Ocean

**DOI:** 10.1038/s41598-021-86043-2

**Published:** 2021-03-24

**Authors:** Camille Hayatte Akhoudas, Jean-Baptiste Sallée, F. Alexander Haumann, Michael P. Meredith, Alberto Naveira Garabato, Gilles Reverdin, Loïc Jullion, Giovanni Aloisi, Marion Benetti, Melanie J. Leng, Carol Arrowsmith

**Affiliations:** 1 CNRS/IRD/MNHN Laboratoire d’Océanographie et du Climat-Expérimentations et Approches Numériques, Sorbonne Université, Paris, France; 2 Atmospheric and Oceanic Sciences Program, Princeton University, Princeton, USA; 3 British Antarctic Survey, Cambridge, UK; 4 School of Ocean and Earth Science, National Oceanography Centre, University of Southampton, Southampton, UK; 5 Institut de Physique du Globe de Paris, Sorbonne Paris Cité, Université Paris Diderot, UMR 7154 CNRS, Paris, France; 6 Institute of Earth Sciences, University of Iceland, Reykjavik, Iceland; 7 NERC Isotope Geosciences Laboratory, British Geological Survey, Nottingham, UK; 8 Centre for Environmental Geochemistry, University of Nottingham, Nottingham, UK

**Keywords:** Physical oceanography, Ocean sciences

## Abstract

The Atlantic sector of the Southern Ocean is the world’s main production site of Antarctic Bottom Water, a water-mass that is ventilated at the ocean surface before sinking and entraining older water-masses—ultimately replenishing the abyssal global ocean. In recent decades, numerous attempts at estimating the rates of ventilation and overturning of Antarctic Bottom Water in this region have led to a strikingly broad range of results, with water transport-based calculations (8.4–9.7 Sv) yielding larger rates than tracer-based estimates (3.7–4.9 Sv). Here, we reconcile these conflicting views by integrating transport- and tracer-based estimates within a common analytical framework, in which bottom water formation processes are explicitly quantified. We show that the layer of Antarctic Bottom Water denser than 28.36 kg m$^{-3}$ $\gamma _{n}$ is exported northward at a rate of 8.4 ± 0.7 Sv, composed of 4.5 ± 0.3 Sv of well-ventilated Dense Shelf Water, and 3.9 ± 0.5 Sv of old Circumpolar Deep Water entrained into cascading plumes. The majority, but not all, of the Dense Shelf Water (3.4 ± 0.6 Sv) is generated on the continental shelves of the Weddell Sea. Only 55% of AABW exported from the region is well ventilated and thus draws down heat and carbon into the deep ocean. Our findings unify traditionally contrasting views of Antarctic Bottom Water production in the Atlantic sector, and define a baseline, process-discerning target for its realistic representation in climate models.

## Introduction

The large-scale ocean overturning circulation distributes climatically-important tracers such as heat, freshwater and carbon around the globe^1^. This global circulation plays an essential role in the planetary climate system. The rate at which water is cycled through the abyssal ocean sets the timescale for interactions between the deep ocean and the atmosphere^2^. The world’s densest water-mass, Antarctic Bottom Water (AABW), sinks to the abyssal ocean near the Antarctic continental margins^3^; its production rate is therefore a key climate variable. AABW production has global impacts on climate on time-scales ranging from decades to millennia, including consequences for sea-level rise^4^, ocean heat content^5^, and large-scale circulation systems such as the Antarctic Circumpolar Current and the Atlantic Meridional Overturning Circulation^6–8^.

In spite of its global climatic relevance, quantification of the rate and underpinning processes of AABW production remains elusive. The largest AABW production site is the Weddell Sea sector of the Southern Ocean, estimated to contribute at least 50% to the total AABW formation^9^. This sector represents one of the most intensively-studied AABW production sites. Estimates of AABW production rates here have been attempted using widely-different methods based on observations of current velocity, hydrography, passive tracers, or isotopes of various chemical elements^10–26^. However, the estimates from this thread of studies remain inconsistent. In this paper, we use a combination of tools from physical and geochemical oceanography to revisit observations spanning over more than 40 years, and quantitatively disentangle the key processes in AABW production. We show that past estimates can be reconciled when robust understanding of how they relate to AABW production is obtained.

AABW formation around the Antarctic continent can be conceptualized as a two-step process. First, interactions between the atmosphere, ocean and cryosphere lead to the formation of extremely cold and relatively well-oxygenated Dense Shelf Water (DSW) on the continental shelves. Second, DSW sinks down the continental slope, entraining warmer Circumpolar Deep Water (CDW), to ultimately form AABW^3,11,27^. While these two mechanisms effectively contribute to the formation of AABW, only the first one actively ventilates the abyssal ocean, with changes in the rates of the two mechanisms dependent on different forcings^3^. Furthermore, ventilation rate of AABW formation is the critical term that needs to be known to understand the exchange rate of carbon-dioxide between the atmosphere and the abyssal ocean. Nevertheless, this complex coastal AABW formation is difficult to represent in global climate models that instead mainly create AABW through a third mechanism; open-ocean deep convection where well-ventilated near surface waters such as Winter Water (WW) sink under strong atmospheric forcing conditions to create AABW by cooling.

**Figure 1:**
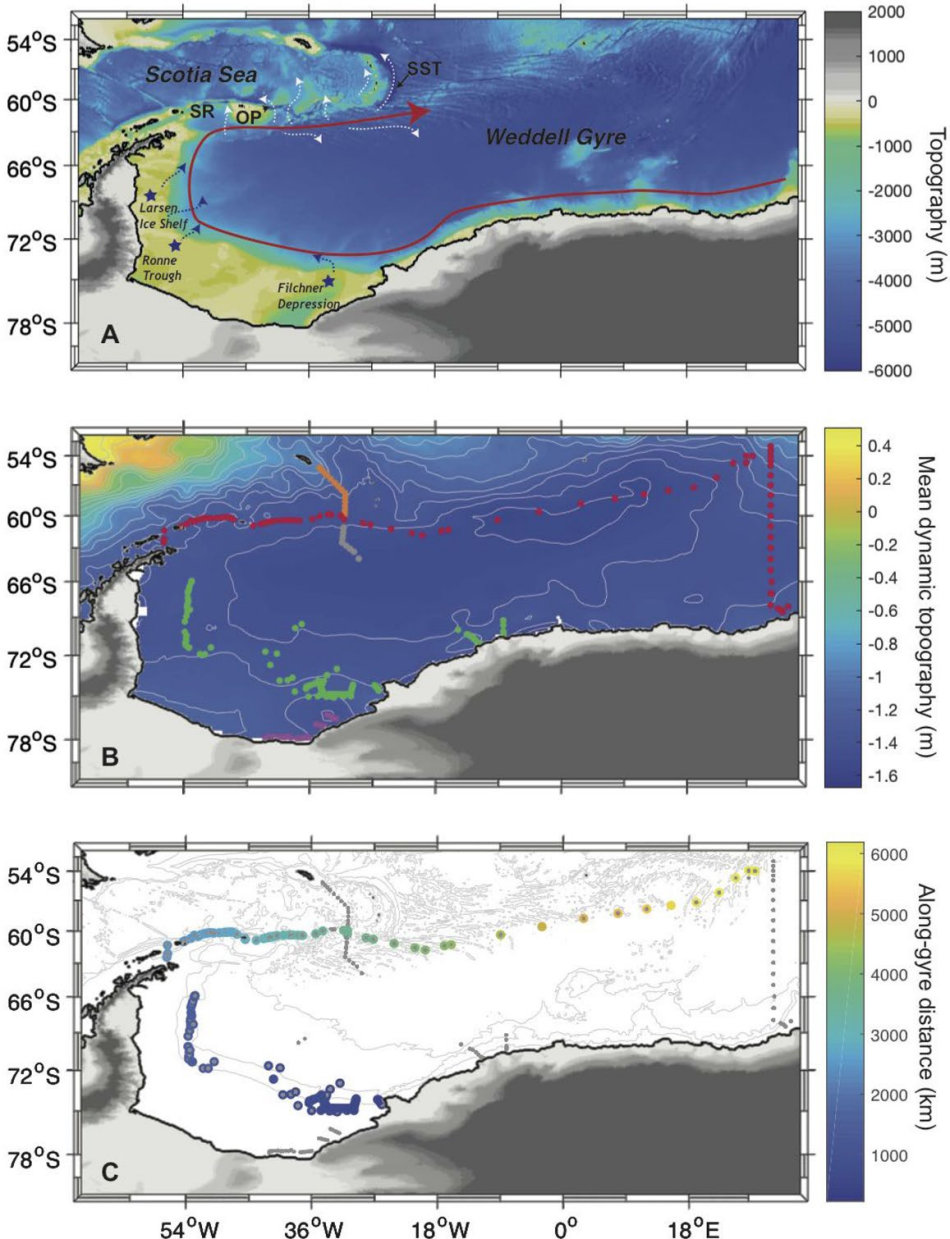
(**A**) Topography in the Atlantic sector of the Southern Ocean. The cyclonic Weddell gyre is schematically indicated by the red arrow. The blue stars and dotted arrows mark the main formation and cascading area of DSW. The dotted white arrows show the AABW export routes in the northern sector of the gyre. From west to east, *SR:* South Scotia Ridge, *OP*: Orkney Passage, *SST*: South Sandwich Trench. (**B**) Map showing the position of the compiled observation database used in this study, with color code corresponding to different dynamical region: (WAPITI observations in purple) continental shelf; (IWSOE 73, Ice Station Weddell, SR02 and WAPITI observations in green) continental slope; (ANDREX/I06S observations in red) Northern and Eastern boundary of the Weddell sector starting in Bransfield Strait to the north of the tip of the Antarctic Peninsula; (gray) southern and (orange) northern part of the A23 repeat section. The background color and contours in gray represent the mean dynamic topography^60^ as an indication of the main circulation pattern in the region. (**C**) Indicative streamwise distance from the Filchner Depression to the northeastern corner of the gyre for all stations distributed along the rim of the gyre (used in Fig. 3). Maps were produced using the software Matlab R2019a.

Local studies using oceanographic data (i.e. geostrophic velocities, CTD profiles, and tracers such as $\delta ^{18}$O and noble gases) have estimated the production rate of DSW cascading off the shelf at different locations along the Antarctic shelf break. As shown in Fig. 1A, the main locations of DSW feeding the deep Weddell basin are the eastern^28^ (i.e. Filchner Depression) and western (i.e. Ronne Trough) sides^11,17^ of the southern continental shelf, as well as the eastern side of the Antarctic Peninsula downstream of Larsen Ice Shelf^14^. At this latter site, cascading waters are either produced locally near Larsen Ice Shelf^15,17,19,29,30^, or originate from the southern shelves but cascade along the western shelves^14^. One of the best-monitored shelf break sites is the Filchner Depression, where the production of DSW has been estimated as 1.6 ± 0.5 Sv^28^. However, inferring a basin-scale flux of DSW ventilating the deep Weddell Sea from the above studies is problematic, because cascades of DSW can occur in plumes flowing in narrow canyons^28^, not all of which are well known or characterised; and large parts of the shelf break are difficult to access and monitor due to persistent sea-ice coverage. Furthermore, such estimates do not provide a quantification of the CDW that is entrained to produce AABW.

Alternatively, basin-scale AABW production rates in the Weddell Sea have been estimated using two main approaches: first, using the distribution of passive tracers from which ventilation rates can be inferred, and (with assumptions) can be converted into bottom water production rates^3,31^ (Supplementary Note 1); second, through mass balance analyses across the entire region^24–26^. While studies using the former approach tend to agree on a Weddell Sea bottom water production rate of 3.7–4.9 Sv^3,31^, studies based on the second approach tend to cluster around a rate approximately twice as large, of 8.4–9.7 Sv^24–26^. Here we explore the causes of this disagreement by estimating the AABW production rate using a mass balance of the southwestern Weddell Sea, in which we use the oxygen isotope composition ($\delta ^{18}$O) of seawater as a tracer that delineates well-ventilated DSW and old CDW, to disentangle their respective contributions to AABW production rate. Water-masses ventilated on the Antarctic continental shelves commonly have low $\delta ^{18}$O associated with meteoric water input (glacial meltwater and precipitation), in contrast with the higher $\delta ^{18}$O of the CDW^32^.

In this study, we compiled an observational dataset of oxygen isotope composition of seawater ($\delta ^{18}$O, see the Methods section). We commence by qualitatively describing the water-mass transformations in the Weddell gyre and the paths currently followed by the densest waters identified on property maps. Then, we decompose AABW into proportions of its original water-mass constituents prior to mixing (referred to as “source” water-masses). Using velocity field estimates obtained from an inversion study of the region^26^, we obtain the net 2008–2010 mean transport of “source” DSW and CDW into and out of the gyre domain. Finally, by quantifying the volumes of the newly-formed DSW and of the CDW entrained downslope, we assess the proportion of AABW formed in the Weddell gyre and the role played by mixing of water-masses near the Antarctic continental margins.

## Results

AABW is defined as the water-mass denser than a neutral density ($\gamma _{n}$) of 28.27 kg m$^{-3}$^3,26,33–35^. It is further decomposed into Weddell Sea Deep Water (28.27 kg m$^{-3}< \gamma _{n} < 28.40\;\hbox { kg m}^{-3}$; WSDW) and a denser variety, Weddell Sea Bottom Water ($\gamma _{n}$ $\ge$ 28.40 kg m$^{-3}$; WSBW^28,36^). WSBW is too dense to flow northward over the ridge system that separates the Weddell gyre from the mid-latitude Atlantic Ocean (the South Scotia Ridge; SR in Fig. 1A). Accordingly, it either escapes the Weddell gyre northward through the South Sandwich Trench (SST in Fig. 1A) along the eastern side of the South Sandwich Islands^37^ or remains within the gyre until it mixes into lighter density classes and becomes WSDW^11,28,36^ (Fig. 1A).

In the southwestern Weddell Sea, which is the focus of this study, the circulation of the gyre corresponds to a preferential conversion of CDW and light WSDW ($28.27\;\hbox { kg m}^{-3}< \gamma _{n} < 28.35\; \hbox { kg m}^{-3}$) and a production of dense WSDW (28.35 kg m$^{-3}$ $\le$ $\gamma _{n} < 28.4\hbox { kg m}^{-3}$) and WSBW^25,26^. We therefore focus here on the dense WSDW and on the WSBW (bottom water with $\gamma _{n}$ $\ge$ 28.35 kg m$^{-3}$) that locally source AABW, and aim at decomposing this bottom water into an admixtures of “source” water-masses to investigate its origin. In contrast to a water-mass that is defined as a loose range of characteristics in $\Theta$–S$_{A}$ (conservative temperature and absolute salinity) or $\gamma _{n}$ spaces, a “source” water-mass is defined as having specific characteristics, representing the properties of the newly-formed water-mass prior to mixing.

We define “source” CDW, using the mean and standard deviation of the observed water-mass characteristics between 28 and 28.27 kg m$^{-3}$ $\gamma _{n}$ and with positive $\delta ^{18}$O values: $\Theta$ = 0.78 ± 0.6 $^{\circ }$C, S$_{A}$ = 34.85 ± 0.04 g kg$^{-1}$, $\delta ^{18}$O = + 0.04 ± 0.03‰ (see “Methods” section). Indeed, CDW entering the Weddell sector (i.e. before it mixes in the Weddell gyre) is an old, poorly-ventilated water-mass, which is clearly distinguished from lower-$\delta ^{18}$O waters associated with high-latitude ventilation processes. In contrast, DSW is associated with very low $\delta ^{18}$O partly resulting from ocean–ice shelf interactions^28,32,36^. The “source” DSW is defined as: $\Theta$ = − 1.99 ± 0.08 $^{\circ }$C, S$_{A}$ = 34.79 ± 0.06 g kg^-1^, $\delta ^{18}$O = − 0.51 ± 0.08‰  and $\gamma _{n}$ = 28.61 ± 0.16 kg m$^{-3}$ (see “Methods” section). Over the Filchner continental shelf, this cold DSW is formed as an admixture of High Salinity Shelf Water (HSSW), glacial meltwater, WW and CDW^32^. CDW feeds the formation of WW and HSSW: both are produced as a result of winter convection under sea-ice but HSSW is associated with local regions of intense brine rejection (e.g. coastal polynyas); HSSW is dense enough to enter the ice-shelf cavities and becomes slightly fresher and cooler as a result of its interaction with the basal ice. We acknowledge that this definition of DSW emphasizes the characteristics of DSW outflowing the Filchner Depression, while other types of DSW cascading elsewhere could have other properties because, for instance, of less or no interaction with ice shelves^11,27^. However, the relatively broad error bars chosen in our definition of DSW allow representation of different types of DSW, including those cascading on the western continental shelves. This assumption appears reasonable from the limited $\Theta$–S$_{A}$ observations obtained on the continental slope directly downstream of the Larsen continental shelf, which suggests that their characteristics are slightly warmer in the Larsen region^38^ but within the standard deviation of DSW found in the Filchner Depression (Supplementary Note 2).

**Figure 2:**
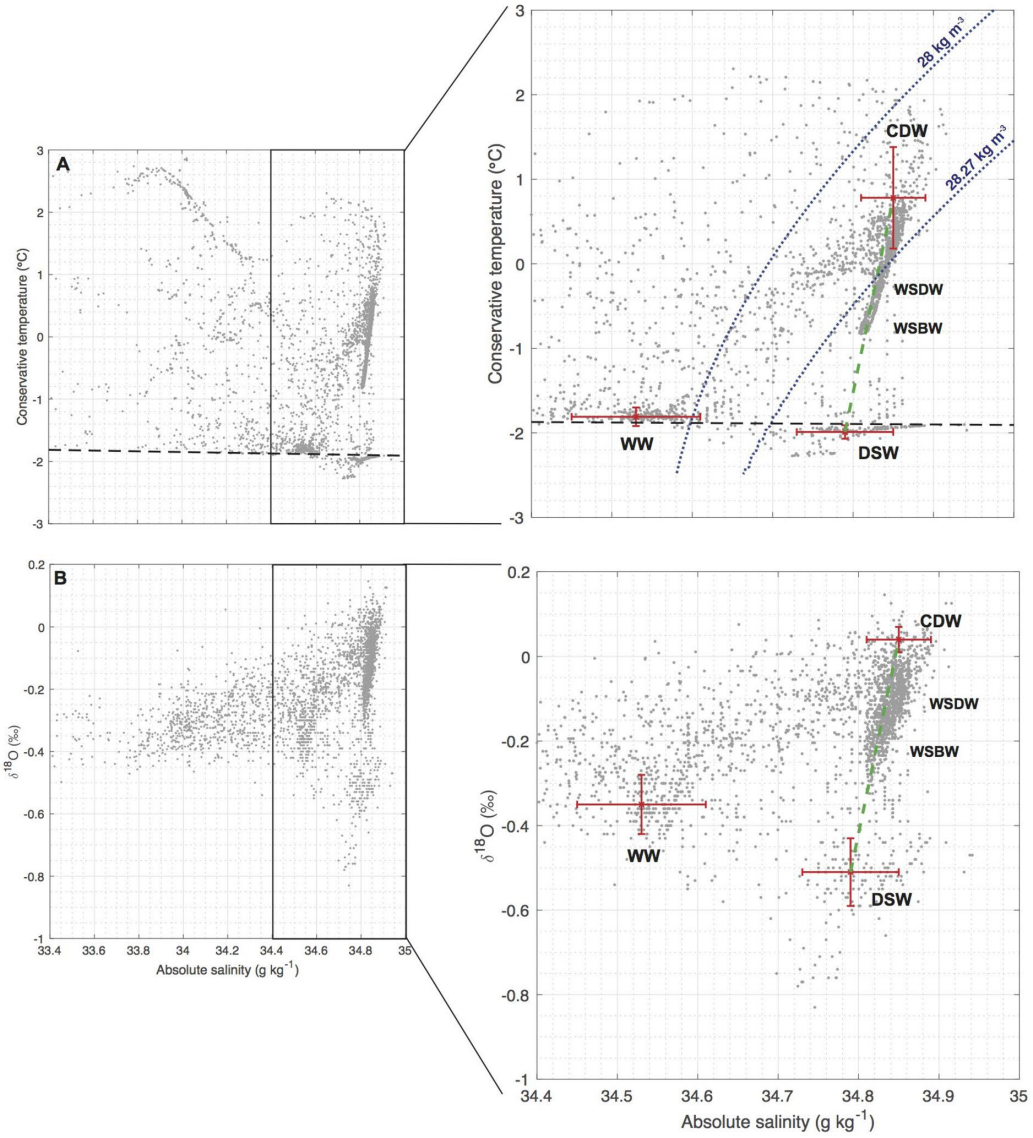
(**A**) $\Theta$-S$_{A}$ and (**B**) $\delta ^{18}$O–S$_{A}$ diagrams showing observations from the compiled dataset used in this study (see Fig. 1B; the Methods section). Neutral density surface 28 and 28.27 kg m$^{-3}$ selected as the CDW interface are superimposed as blue dashed curves in panel A; and surface freezing line as black dashed line. Mean and standard deviation of “source” water-masses (CDW, DSW and WW) characteristics are indicated as red crosses.

On $\Theta$–S$_{A}$ and $\delta ^{18}$O–S$_{A}$ diagrams of the historical profiles of the region, WSDW and WSBW appear clearly on a straight and well-defined line bounded by CDW and DSW characteristics (Fig. 2). This is consistent with the prevalent view that they are formed from DSW cascading off the continental shelves and mixing with CDW by entrainment. This alignment in both parameter spaces supports our definition of “source” water-masses, including the definition adopted for DSW. The $\Theta$–S$_{A}$ and $\delta ^{18}$O–S$_{A}$ diagrams suggest that lighter water-masses mix with a third “source” water-mass, WW, defined as: $\Theta$ = –1.81 ± 0.1 $^{\circ }$C, S$_{A}$ = 34.53 ± 0.08 g kg$^{-1}$, $\delta ^{18}$O = –0.35 ± 0.07‰  and $\gamma _{n}$ = 28 ± 0.14 kg m$^{-3}$ using mean and standard deviation of the characteristics observed at the temperature minimum in subsurface on the southern Weddell Sea continental shelf (see the Methods section).

**Figure 3:**
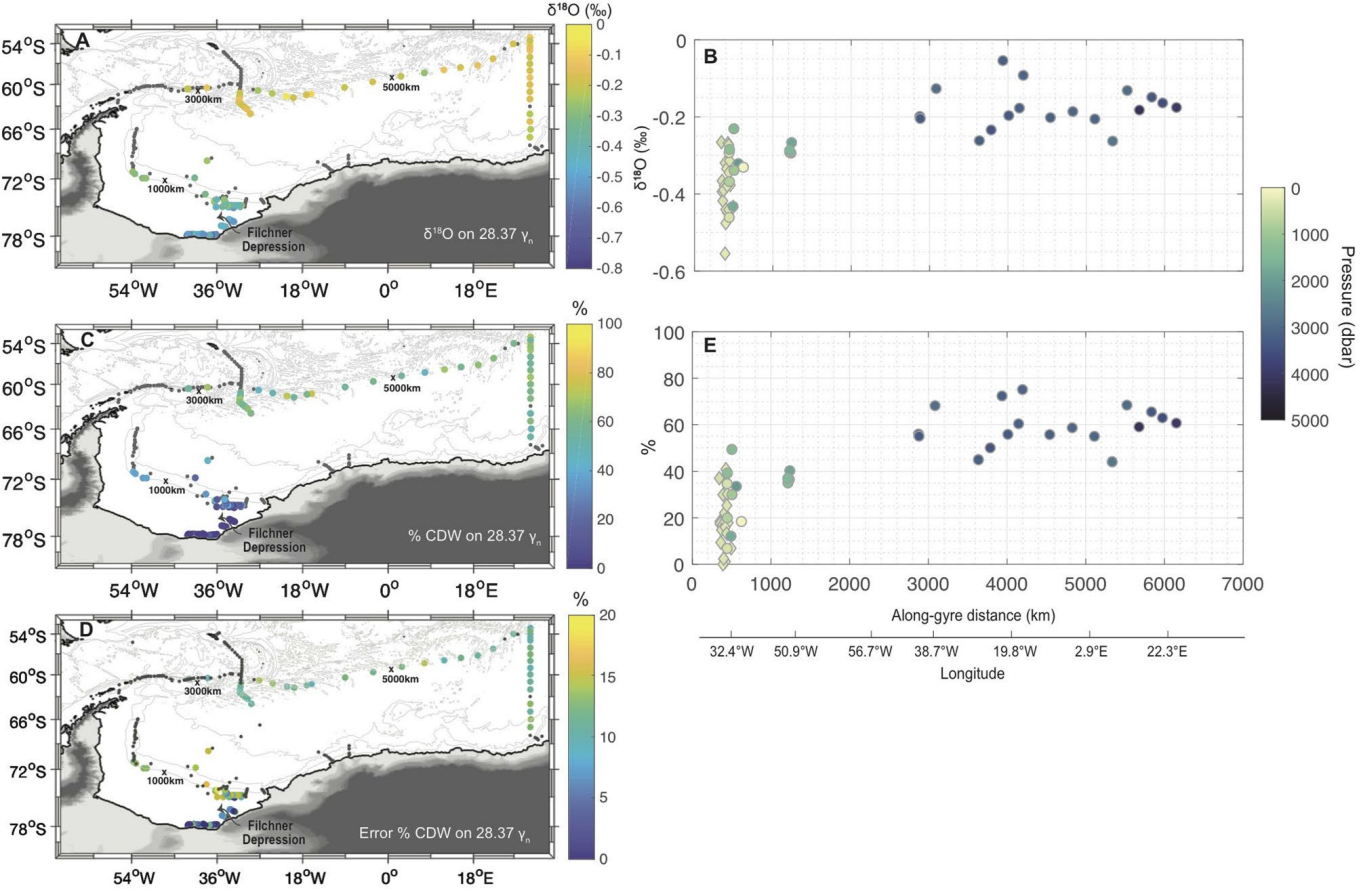
(**A**) Spatial maps of $\delta ^{18}$O on the 28.37 kg m$^{-3}$ $\gamma _{n}$ surface, and (**B**) corresponding stream-wise change of $\delta ^{18}$O along the rim of the gyre from the Filchner Depression (origin of the along-gyre distance in abscissa) to the northeastern corner of the gyre (see Fig. 1C). (**C**) Spatial maps of fraction of CDW and its corresponding error (**D**) on the 28.37 kg m$^{-3}$ $\gamma _{n}$ surface, and (**E**) corresponding to stream-wise change of % of CDW along the rim of the gyre from the Filchner Depression to the northeastern corner of the gyre. Maps were produced using the software Matlab R2019a.

Spatial mapping of the $\delta ^{18}$O characteristics of bottom water is useful in understanding how they are influenced by low-$\delta ^{18}$O DSW formed on continental shelves. For each profile of the historical dataset, we compute the $\delta ^{18}$O interpolated linearly onto the 28.37 kg m$^{-3}$ $\gamma _{n}$ surface (Fig. 3A). On the continental shelf in the Filchner Depression (75–78 $^{\circ }$S), the 28.37 kg m$^{-3}$ $\gamma _{n}$ surface is associated with very depleted $\delta ^{18}$O values, as a result of the influence of glacial meltwater in this region^32^. As this water-mass flows out along the continental slope following the clockwise circulation of the gyre, it quickly loses its $\delta ^{18}$O signature (Fig. 3A), consistent with rapid mixing with overlying $\delta ^{18}$O-enriched water. On the northern side of the gyre, the $\delta ^{18}$O characteristics of 28.37 kg m$^{-3}$ $\gamma _{n}$ surface stabilize around − 0.2‰, suggesting that mixing is less intense away from the continental margins (Fig. 3B). We note that this gyre-scale structure does not stem from sampling over a large temporal range (1973–2017), as interannual variability within each water-mass in $\Theta$–S$_{A}$ and $\delta ^{18}$O–S$_{A}$ spaces is much smaller than this observed spatial variability (Supplementary Note 3). Away from the continental shelf, the 28.37 kg m$^{-3}$ $\gamma _{n}$ surface is only present in the deepest part of the gyre, corroborating the trapping of dense WSDW and WSBW within the gyre. Superimposed on this gyre-scale signal, we also observe local variability. Although we cannot exclude that this local variability and its spatial pattern may be explained by sampling bias, we note that there are a variety of different processes that might be responsible, including distinct source waters cascading downslope and differing local mixing.

We now use the $\delta ^{18}$O observations in combination with other tracers to quantify the mixed fraction of different “source” water-masses composing each water-parcel on the 28.37 kg m$^{-3}$ $\gamma _{n}$ surface (Methods section; Eq. 1). We decompose water-parcels into a mixture of CDW, DSW, and WW. We note that linear combination of only these three endmembers can explain most of the conservative temperature-absolute salinity characteristics of the Weddell gyre (Supplementary Note 4). Errors on the fractions are computed as standard deviation of 1000 Monte-Carlo experiments, in which we repeat the same decomposition, but adding a random perturbation to the definition of each “source” water-mass characteristics within a range of their defined error bars (see the Methods section). A general south-to-north increase in the CDW fraction (Fig. 3C) with its corresponding error (Fig. 3D) is assessed on the 28.37 kg m$^{-3}$ $\gamma _{n}$ surface, consistent with the previously-discussed increase in $\delta ^{18}$O. The fraction of CDW increases rapidly along the continental slope from the southern Filchner Depression to the northern tip of the Antarctic Peninsula and stabilizes away from the continental margins. The increase is substantial, from 0 to 60% in the gyre interior away from the continental margins (Fig. 3E). Even away from the neutral density boundary of the CDW (i.e 28 kg m$^{-3} \le \gamma _{n}$ $\le$ 28.27 kg m$^{-3}$), the 28.37 kg m$^{-3}$ $\gamma _{n}$ surface still contains about 60% of CDW away from the continental slope, implying intense mixing activity near the slope.

**Figure 4:**
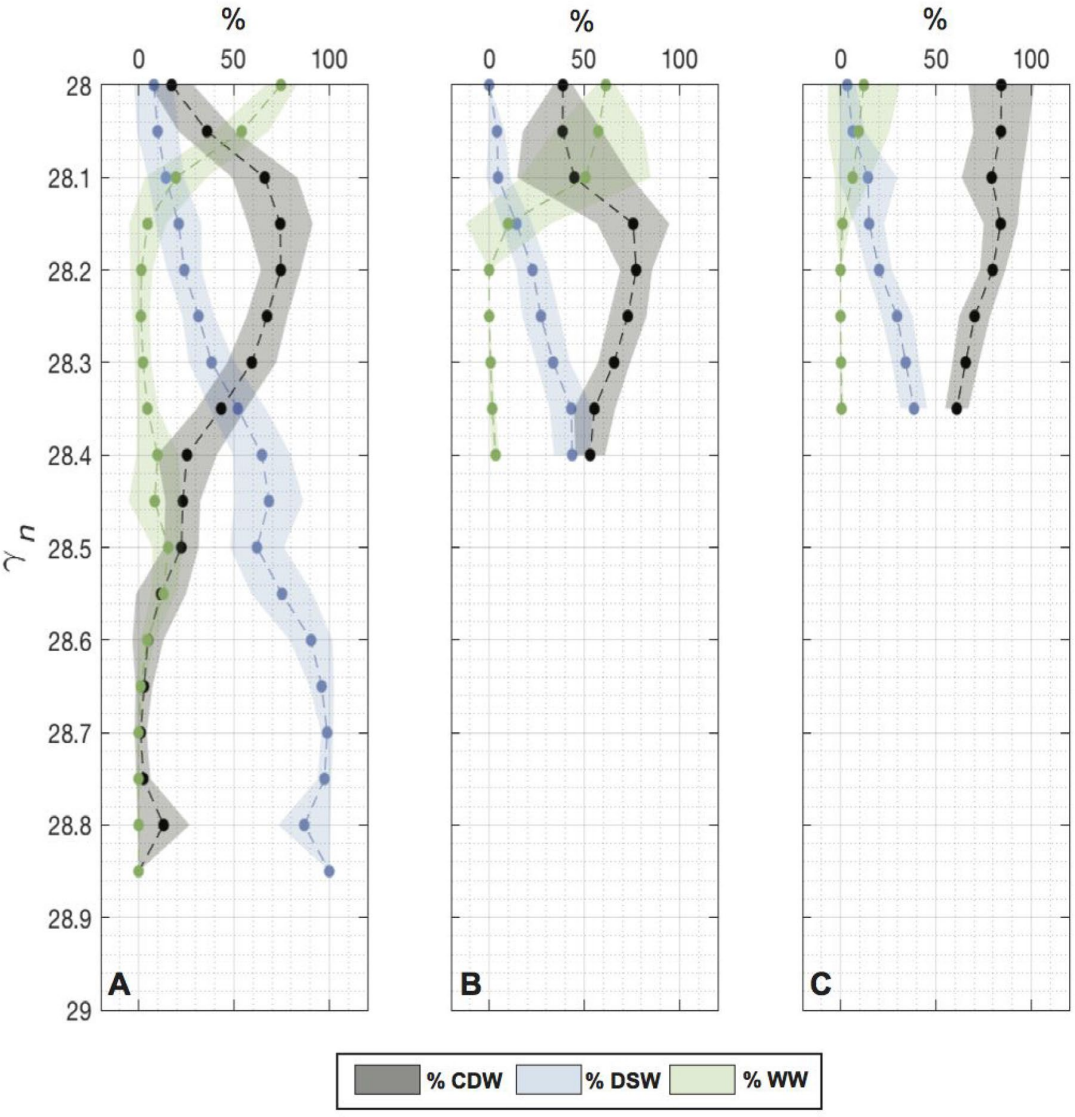
Mean percentage contributions of “source” water-masses (green: WW, blue: DSW and black: CDW) in the Weddell gyre along (**A**) the continental slope (green stations in Fig. 1B), (**B**) the gyre side of the A23 segments (gray stations in Fig. 1B) and (**C**) the Scotia Sea side of the A23 segment (orange stations in Fig. 1B). The mean percentage contributions are computed in 0.025 $\gamma _{n}$ bins. Shading indicates standard deviations around the mean of each $\gamma _{n}$ bins. Standard deviation ranges include negative and above 100% water-masses fraction, these are however not physically plausible solutions.

The vertical structure of the admixture can be further examined by showing the fractions of “source” water-masses in discrete $\gamma _{n}$ layers, averaged for different sectors of the gyre: the continental slope sector (Fig. 4A; corresponding to mean fractions of stations shown as green dots in Fig. 1B); the gyre side of A23 section (Fig. 4B; corresponding to mean fractions of stations shown as gray dots in Fig. 1B); and the Scotia Sea side of A23 section (Fig. 4C; corresponding to mean fractions of stations shown as orange dots in Fig. 1B). Clearly, on the continental slope, the bottom water ($\ge$ 28.35 kg m$^{-3}$ $\gamma _{n}$) is mostly composed of DSW, ranging between 60 and 100% for layers denser than 28.5 kg m$^{-3}$ $\gamma _{n}$ (Fig. 4A). In contrast, layers lighter than 28.35 kg m$^{-3}$ $\gamma _{n}$ are strongly dominated by a CDW signature, with proportions around 65% and a contribution of WW ranging from 20 to 75% for waters with $\gamma _{n}$ $\le$ 28.1 kg m$^{-3}$ (Fig. 4A). Comparing this “source” water fraction distribution with its counterpart on the northern side of the gyre (Fig. 4B,C) clearly indicates that CDW and DSW have actively mixed in the bottom water layers ($\ge$ 28.35 kg m$^{-3}$ $\gamma _{n}$), echoing our interpretation of Fig. 3. Interannual variability of endmember water-mass characteristics is mostly confined within the error bars of our endmember definitions (Supplementary Note 3), which ensures that our results are not a reflection of temporal variability but indeed manifest a robust gyre-scale structure.

In addition, a quantitative examination of the A23 transect reveals large differences between the southern and northern sides of the section for a given neutral density class. The southern side of A23 is inside the Weddell gyre; at this location, and consistent with our preceding analysis, bottom water at 28.35–28.4 kg m$^{-3}$ $\gamma _{n}$ is composed of nearly equal proportions of DSW and CDW (Fig. 4B). Conversely, on the northern side of A23 in the Scotia Sea, the 28.35 kg m$^{-3}$ $\gamma _{n}$ density level is marginally more dominated by the CDW contribution (Fig. 4C). The increased contribution of CDW in the bottom water of the Scotia Sea suggests that the flow over relatively shallow passages of the South Scotia Ridge^24,39^ (e.g. Orkney Passage) has generated significant mixing^40^.

**Figure 5:**
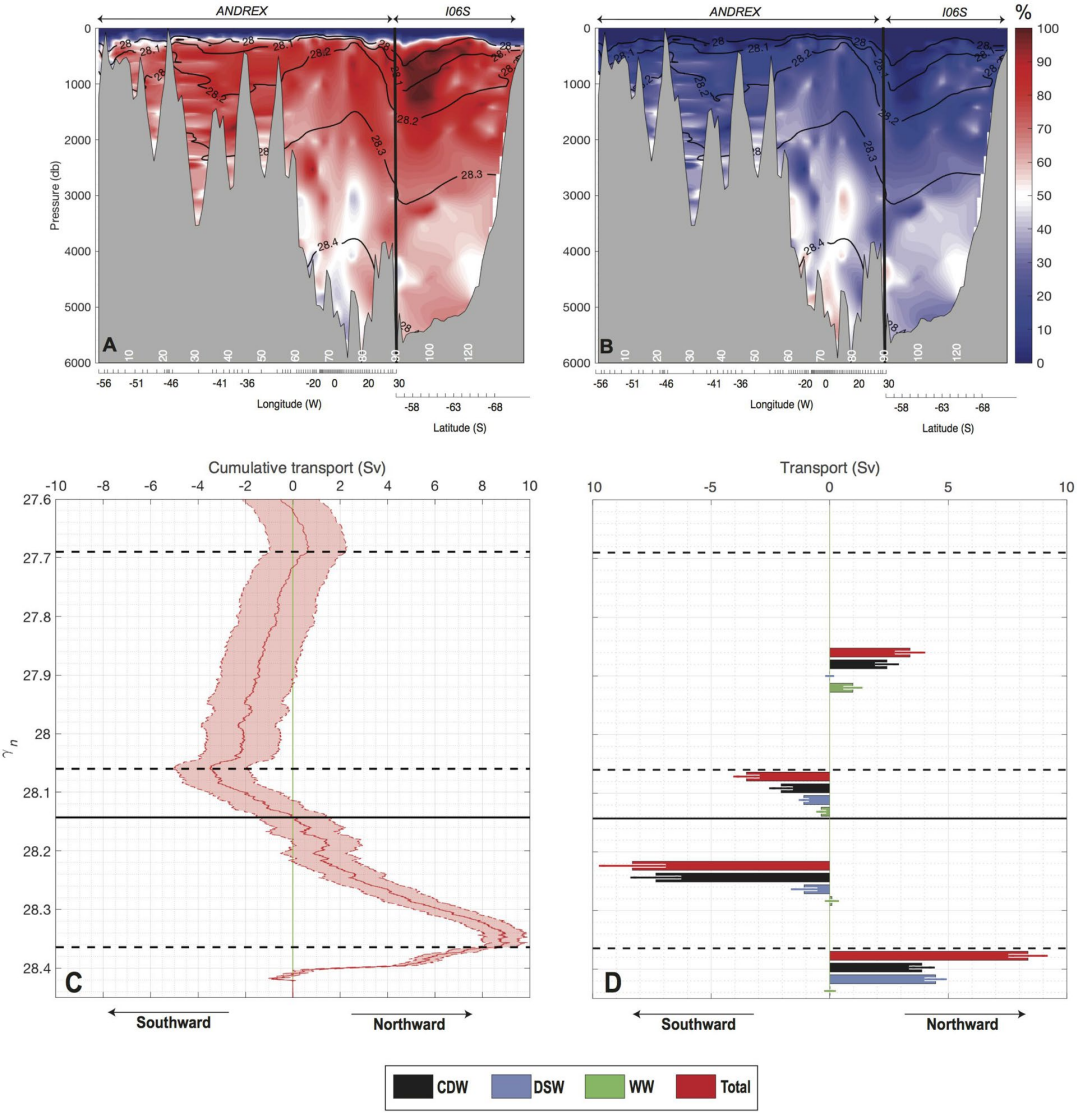
Percentage contributions of (**A**) CDW and (**B**) DSW across the ANDREX/I06S section (red stations in Fig. 1B). Black contours indicate neutral density isopycnals and x-axis is based on uneven linear longitude/latitude axis corresponding to station numbers in white. (**C**) Total cumulative transport from the bottom ocean upward, summed across the ANDREX/I06S section, computed in neutral density coordinates. Shading indicates errors from a Monte-Carlo error estimate. Positive (negative) transport are directed out of (into) the gyre. (**D**) Net transports across the ANDREX/I06S into four density layers: 27.69–28.06 kg m$^{-3}$ $\gamma _{n}$; 28.06–28.14 kg m$^{-3}$ $\gamma _{n}$; 28.14–28.36 kg m$^{-3}$ $\gamma _{n}$; and denser than 28.36 kg m$^{-3}$ $\gamma _{n}$. For each of these layers, the total net transport (red bars) are decomposed into their end-member contributions: (black) CDW, (blue) DSW, (green) WW. Positive (negative) transport are directed out of (into) the gyre.

The A23 section indicates that some DSW escapes the Weddell gyre as part of the northward export of bottom water. One can quantify the net DSW export away from the gyre. Jullion et al.^26^ estimated the net water-mass transport across the ANDREX/I06S section, shown as red dots in Fig. 1B, by adjusting geostrophic velocities across the section with an inverse model performed using observations spanning 2008– 2010. Here we use these estimates of adjusted geostrophic velocities to estimate the net DSW, CDW and WW transports across the ANDREX/I06S section, representative of a 2008-2010 mean (Supplementary Note 3). Fractions of CDW and DSW along the section are shown in Fig. 5A,B respectively. In the deep ocean, the influence of WW is very weak so that the contributions of DSW and CDW add up to 100% for waters denser than 28 kg m$^{-3}$ $\gamma _{n}$; WW makes a significant contribution only for the layer lighter than 28 kg m$^{-3}$ $\gamma _{n}$. We note that our estimates of fractions of “source” water-masses are unreliable near the surface (shallower than approximately 200 m depth), because any meteoric inputs and surface freshwater fluxes (e.g. precipitation/evaporation, glacial meltwater and/or local sea-ice melting/freezing) in addition to those occurring on the continental shelves (which are inherently included in the DSW definition) are not accounted for in this layer in Eq. (1). We thus focus our analysis in the deep layers (typically denser than 28 kg m$^{-3}$ $\gamma _{n}$), where our approach is most reliable.

The highest percentage of CDW is found between 300 and 1200 dbar (Fig. 5A), with a maximum in the northeastern corner of the ANDREX/I06S section, where the Antarctic Circumpolar Current crosses the corner twice (entering and leaving the domain defined by the ANDREX/I06S section), as shown by the dipping and heaving of the isopycnal surfaces (Figs. 1A, 5A,B). As expected, the highest percentage of DSW is found in the bottom layers (Fig. 5B), where it makes a contribution to the volume of AABW approximately equal to that of CDW. Based on this decomposition and collocated Jullion et al.^26^’s adjusted geostrophic velocities, the total cumulative transport from the bottom of the ocean ocean can be derived (Fig. 5C). From the ocean floor toward lighter layers, the cumulative transport increases, reflecting a northward transport of the densest layer, denser than 28.36 kg m$^{-3}$ $\gamma _{n}$ (Fig. 5C). In waters of density from 28.36 kg m$^{-3}$ $\gamma _{n}$ to 28.06 kg m$^{-3}$ $\gamma _{n}$, the cumulative transport decreases, reflecting a southward transport of this density range. Then, the lighter layer from 28.06 kg m$^{-3}$ $\gamma _{n}$ to 27.69 kg m$^{-3}$ $\gamma _{n}$ displays a northward transport (Fig. 5C). The cumulative transport from the ocean floor across the section reflects therefore a two-celled overturning structure, with an inter-cell density boundary, defined as the density where the cumulative transport integrated from the seafloor crosses zero, located at 28.14 kg m$^{-3}$ $\gamma _{n}$^25^ (horizontal black line in Fig. 5C). Using the decomposition into “source” water-masses, we estimate the net transport of DSW and CDW within the lower cell (denser than 28.14 kg m$^{-3}$ $\gamma _{n}$), which is fed by a southward flow in the density range 28.14–28.36 kg m$^{-3}$ $\gamma _{n}$, comprising an admixture of 7.3 ± 0.9 Sv of CDW and 1.1 ± 0.4 Sv of DSW (Fig. 5D). This is converted into denser bottom water outflowing northward in water denser than 28.36 kg m$^{-3}$ $\gamma _{n}$ as an admixture of 3.9 ± 0.5 Sv of CDW and 4.5 ± 0.3 Sv of DSW (Fig. 5D). The Weddell Sea-sourced AABW ($\gamma _{n} > 28.36\; \hbox { kg m}^{-3}$^25,26^), thus results from the conversion by continental shelf processes of 3.4 ± 0.6 Sv of CDW into DSW ventilating the deep ocean, and the entrainment of 3.9 ± 0.5 Sv of CDW mixing diapycnally into the dense plume cascading down the southwestern continental slopes. One notes that WW does not contribute to Weddell Sea-sourced AABW showing that open-ocean deep convection might not be a suitable process for present day AABW formation in this region, in contrast with what is commonly simulated in global climate models^41^.

## Discussion

The complex processes governing the AABW formation in the Weddell basin have been quantified using $\delta ^{18}$O of seawater on the southern continental shelf, along with hydrographic transects at the northern edge of the gyre. We have disentangled the rate of production of DSW and the admixture of CDW by entrainment, which ultimately replenish the AABW exported to the world’s ocean.

At the gyre scale, the $\delta ^{18}$O distribution of water-masses on the 28.37 kg m$^{-3}$ $\gamma _{n}$ surface provides insight into the ventilation of bottom water and its spreading patterns. The spatial distribution of $\delta ^{18}$O on the 28.37 kg m$^{-3}$ $\gamma _{n}$ surface reflects the influx of low-$\delta ^{18}$O ventilated waters on the continental slope, quickly losing their signature as they mix with ambient overlying CDW along their pathway in the cyclonic Weddell gyre. Their characteristics stabilize on the northern side of the gyre, away from the continental slope. Consistent with this, we find that the 8.4 ± 0.7 Sv of AABW denser than 28.36 kg m$^{-3}$ $\gamma _{n}$ that is exported northward from the Weddell Sea in 2008-2010^25,26^ is composed of 4.5 ± 0.3 Sv of DSW, of which 3.4 ± 0.6 Sv is newly formed in the domain of the southwestern continental shelves, and 3.9 ± 0.5 Sv of CDW that mixes diapycnally into the dense plume.

We provide an advanced understanding of AABW production rates in the Weddell Sea. By combining a mass balance approach with a ventilation tracer approach in a single framework, we resolve a conundrum that has remained in previous studies: 8.4 ± 0.7 Sv of AABW denser than 28.36 kg m$^{-3}$ $\gamma _{n}$ is fully consistent with estimates of 4–5 Sv obtained from ventilation tracer studies^3,31^, as those estimates excluded or underestimated entrainment (Supplementary Note 1). In addition, although not always directly comparable because of a slightly different definition of water-masses and/or definition of outflow region, our overall estimate of 8.4 ± 0.7 Sv of AABW production in the Weddell Sea sector is consistent within uncertainties on previous estimates based on inverse models or general circulation models^24,42,43^. Further, we estimate that 3.4 ± 0.6 Sv of DSW is formed on the Weddell Sea continental shelves, which accords well with previous local estimates on the continental sill and slope. While these estimates are necessarily local, we can extract an overall estimate on a basin-wide scale: about 1.6 ± 0.5 Sv of waters below the freezing point have been estimated to outflow from the Filchner Depression^28,44^; there is some evidence that the two sources of DSW from the Filchner Depression and the western side of the southern continental shelf are roughly equivalent^17^; the relative contribution of DSW production from the Larsen Ice Shelf has been estimated to be about a quarter of that from the Filchner Depression, i.e. $\sim$ 0.4 Sv^30^. A compilation of these studies would therefore produce a net Weddell production of DSW in the range of O(3–4 Sv), again, very much in line with our estimate of 3.4 ± 0.6 Sv.

Once DSW cascades the continental slope, it entrains above-lying CDW, which contributes to further AABW formation. A large body of theoretical work describes potential mixing mechanisms associated with dense plume overflow. Plumes of DSW cascading downslope can attain high speed, with regime transition from supercritical to subcritical speed regimes associated with abrupt changes of the bottom topography and resulting in strong mixing due to hydraulic jumps^28,45^. Other mechanisms can enhance entrainment, including the development of Kelvin–Helmholtz instabilities or roll-like waves, which break and cause vertical mixing, or the development of eddies through barotropic or baroclinic instabilities, in which mixing may occur primarily through lateral stirring processes^46,47^. Taken together, these entrainment mechanisms have been assessed empirically both in models and from observations over the Weddell Sea continental slope and found to potentially increase the initial volume flux of DSW by a factor of 2–3^28,45,48,49^. Here, we provide an observation-based estimate at a basin scale that the volume flux of DSW formed locally on the continental shelves (3.4 ± 0.6 Sv) increases by a factor 2.1 ± 0.7 after the entrainment process occurs on the slope. While many processes could impact AABW formation and transport under climate change^41,50,51^, our results highlight that high-latitude Southern Ocean changes at the surface could have disproportionately strong effects in the global deep ocean and its overturning circulation.

The continental slope between the 600 and 3000 m isobaths, from the Filchner Depression to the tip of the Antarctic Peninsula, covers an area of about 240 $\times$ 10$^{3}$ km$^{2}$. Assuming that all the entrainment of CDW occurs uniformly over the southwestern continental slope, which appears qualitatively consistent with the basin-scale regional distribution of $\delta ^{18}$O (Fig. 3A), we infer that our estimated CDW entrainment would be associated with an averaged diapycnal velocity on the order of $\sim$ 139 ± 17 cm day$^{-1}$ across the 28.36 kg m$^{-3}$ $\gamma _{n}$ layer, which translates into a mean diapycnal diffusion of $\sim$ 1.4 ± 0.2 $\times$ 10$^{-3}$ m$^{2}$ s$^{-1}$, considering the vertical gradient and curvature of the average neutral density profile over the continental slope (Supplementary Note 5). Whilst there are large uncertainties associated with this calculation, we consider it as indicative that very high levels of diapycnal mixing (two orders of magnitude larger than typical background diapycnal mixing rates in the ocean^52^) extend over the southwestern continental slope of the Weddell basin and are associated with the production of Weddell-sourced AABW.

Our analysis demonstrates that the ventilation of the abyss in the Atlantic sector of the Southern Ocean occurs at approximately half the rate of AABW production rate, with the other half of AABW being old CDW entrained on the slope. These are important results in the context of furthering our understanding of heat and carbon uptake and storage in the Southern Ocean^53^, as well as observed contemporary changes of temperature, salinity, thickness, and oxygenation of AABW^4,5,33,54^. Present climate models vary widely in their ability to represent bottom water properties^55–57^ because of important flaws in their representation of bottom water formation processes^56,58^. Because of its importance for ocean-ice shelf interaction, as well as heat and carbon storage^53^, the inadequate representation of the high latitude Southern Ocean in climate models represents an important limitation in our understanding and prediction of future climate. Our observations and physical interpretation provide a target for future model improvements.

## Materials and methods

### Data

The core dataset of this analysis consists of observations from several oceanographic surveys in the Weddell gyre region (Fig. 1B) between 1973 and 2017 that sampled seawater for oxygen isotope analysis among others hydrographic parameters (Table 1). The corresponding streamwise distance from the Filchner Depression to the northeastern corner of the gyre for all observations is shown in Fig. 1C. The ANDREXs and I06S sections are merged into one transect extending from the tip of the Antarctic Peninsula to the Antarctic coast at 30$^\circ$E and referred as the ANDREX/I06S section. The set of variables measured along these transects includes physical hydrographic properties (temperature, salinity, and pressure) and $\delta ^{18}$O (as a freshwater tracer) as well as dissolved oxygen. This requires assembling datasets based on measurements made by different groups using varying analytic approaches, and from different years. This is achieved by adjusting the datasets after inter-laboratory and inter-cruise comparison (see Supplementary Note 6 for a description of the adjustments applied). In addition, the analysis includes geostrophic velocity retrievals for the ANDREX/I06S section^26^.

**Table 1:** Summary of the cruise datasets used in this study. *N/A* information not available.

Year	Cruise number	Section name	Location	References
1973	N/A	IWSOE 73	$\sim$ 70$^\circ$S – $\sim$40$^\circ$W	Weiss et al.^12^
1989	ANT-VIII/2	SR02	$\sim$ 70$^\circ$S – $\sim$ 10$^\circ$W	Mackensen et al.^61^
1992	N/A	Ice Station Weddell	$\sim$ 59$^\circ$W along the continental slope in front of the Larsen Ice Shelf	Weppernig et al.^17^
1995	ANT-XII/3	SR02	$\sim$ 73$^\circ$S – $\sim$ 30$^\circ$W	Mackensen et al.^62^
2008	33RR20080204	I06S	$\sim$ 30$^\circ$E between South Africa and Antarctica	Jullion et al.^26^
2009	JC30	ANDREX	$\sim$ 60$^\circ$S between the Antarctic peninsula and $\sim$ 19$^\circ$W	Jullion et al.^26^
2010	JR239	ANDREX	$\sim$ 60$^\circ$S between $\sim$ 19$^\circ$W and $\sim$ 30$^\circ$E	Jullion et al.^26^
2016	JR15006	A23	$\sim$30$^\circ$W between the Orkney Passage and the South Sandwich Trench	Meijers et al.^63^
2017	JR16004	A23	$\sim$ 30$^\circ$W between the Orkney Passage and the South Sandwich Trench	Sallée et al.^64^
2017	JR16004	WAPITI	$\sim$ 76$^\circ$S – $\sim$ 36$^\circ$W in the Filchner Depression	Akhoudas et al.^32^

### Mass balance calculation

To quantify the different water-mass sources that compose the deep and bottom layers of the Weddell gyre, we solve the following three-components mass balance:

1 $$\begin{aligned} \left\{ \begin{array}{lll} 1 = f_{DSW}+f_{CDW}+f_{WW}\\[10pt] S_{A}^{obs} = f_{DSW}\cdot S_{A}^{DSW}+f_{CDW}\cdot S_{A}^{CDW}+f_{WW}\cdot S_{A}^{WW}\\[10pt] \delta ^{18}O^{obs} = f_{DSW}\cdot \delta ^{18}O^{DSW}+f_{CDW}\cdot \delta ^{18}O^{CDW}+f_{WW}\cdot \delta ^{18}O^{WW} \end{array} \right. \end{aligned}$$

with *f*$_{DSW}$, *f*$_{CDW}$, *f*$_{WW}$ and *S*$_{A}$; fraction of DSW, fraction of CDW, fraction of WW and absolute salinity, respectively.

The assumption of steady mean values for “source” endmembers is important in obtaining realistic percentage fraction to a water sample. We consider DSW to be an admixture of High Salinity Shelf Water, Ice Shelf Water, Winter Water and Warm Deep Water formed on the continental shelf. The composition of DSW is determined based on the 2017 WAPITI cruise data^32^, which represents the variety of DSW formed in the Filchner Depression. The properties of this water-mass are thus 34.79 g kg$^{-1}$ for absolute salinity and − 0.51‰ for $\delta ^{18}$O. For CDW, we have chosen its physical properties based on the ANDREX/I06S section with neutral density values of 28 kg m$^{-3}$ and 28.27 kg m$^{-3}$ selected as the CDW upper and lower boundaries. In addition, we limit the CDW domain to $\delta ^{18}$O $\ge$ 0‰ in order to define a CDW layer characterized by the highest $\delta ^{18}$O values^59^. The WW characteristics are based on the WAPITI observations and are defined as the mean and standard deviation of properties found in the temperature minimum layer below the mixed-layer depth on the southern Weddell Sea continental shelf. For CDW and WW, we retain the mean values of 34.85 ± 0.04 g kg$^{-1}$ and 34.53 ± 0.08 g kg$^{-1}$, respectively, for absolute salinity, and 0.04 ±0.04‰ and − 0.35 ± 0.07‰, respectively, for $\delta ^{18}$O. These values represent local varieties of CDW that enter and form in the gyre, and of WW that forms in the southern Weddell Sea. Both of these “source” water-masses are entrained along the continental slope to form the Weddell-sourced AABW. Nonetheless, they are not representative of “source” CDW found further north in the Antarctic Circumpolar Current or “source” WW formed during the previous winter preceding the cruise (WW observed during summer cruises has probably been transformed by vertical mixing since its formation during the previous winter). Oxygen isotope observations cover the Weddell Sea continental shelf and close a section coast to coast (i.e. ANDREX/I06S section) allowing to use it alongside a mass balance calculation.

### Transport of “source” water-masses

Net transport, T, of a “source” water-mass across the ANDREX/I06S section is computed as follows:

2 $$\begin{aligned} T(\gamma _n) = \sum _{k}^{l} \sum _{j}^{m} C [f_{k,j}, ..., u_{k,j},...], \end{aligned}$$

where C = f$_{k,j}\times$ u$_{k,j}\times {\mathcal {A}}_{k,j}$; k = 1, ..., l and j = 1, ..., m are respectively the number of vertical levels and the number of stations at the neutral density $\gamma _n$; ${\mathcal {A}}_{k,j}$ is the area defined by vertical spacing and station spacing; f$_{k,j}$ is the fraction of the “source” water-mass estimated at station j, level k; and u$_{k,j}$ is the geostrophic velocity perpendicular to the section (positive is outward from the southwestern Weddell Sea sector, i.e. mostly northward for the ANDREX section and eastward for the I06S section), which has been adjusted with a basin-scale box inverse model that ensures mass balance across the ANDREX/I06S section, as presented in Jullion et al.^26^. Vertical spacing is computed in density space, using a regular neutral density grid with of step of 0.0001 kg m$^{-3}$ and the transport calculation is done at mid distance between each pair of stations of the ANDREX/I06S section.

### Sources of error

Trying to decompose water-parcels characteristics into source constituents using Eq. (1) can present a number of issues, which we investigate here. First, choosing fixed source water characteristics to decompose observed water-parcels spanning more than 40 years might be inappropriate if source waters have large temporal variability in their characteristics. We analyze this source of error in Supplementary Note 3 and show that the interannual variability of source endmembers is contained within our defined error bars for each source endmember. Second, a linear combination of only three endmembers might not be able to explain the full complexity of the Weddell Sea water-masses. We investigate this in Supplementary Note 4 and show that the careful (physically-based) choice of the three endmembers allows us to explain about 96% of the Weddell Sea sector water-mass volume, the only water-mass not well-represented being in the surface layer and thus is not relevant for our study. Third, errors can originate in propagation of uncertainties of the source endmembers definition, and also intrinsic errors associated with the choice of resolving the question of source constituents using Eq. (1). We investigate the former source of error using a Monte-Carlo experiment where we repeat the resolution of Eq. (1) 1000 times, but slightly modify the source endmembers definition (within their defined error bars). From these 1000 realizations, we use the 80% probability range (10–90% percentile range) as error bars for our estimated fractions. The intrinsic source of error due to the choice of the system of equations itself is harder to estimate, but we approach it by predicting the temperature and dissolved oxygen that the decomposition from Eq. (1) suggests it should be, and then compare this prediction with the observed value (Supplementary Note 7). We show that both temperature and dissolved oxygen can be accurately predicted by the decomposition in waters denser than 28 kg m$^{-3}$ $\gamma _{n}$, within $\sim$ 10% of their respective observed range, which overall provide great confidence in the decomposition provided by Eq. (1).

From Eq. (2), errors on the transport are propagated from the errors on the adjusted geostrophic velocity u$_{k,j}$; $\epsilon _{u_{k,j}}$ obtained as an output of Jullion et al.^26^ inverse model solution and the errors on the fraction of the “source” water-mass f$_{k,j}$; $\epsilon _{f_{k,j}}$ obtained from the 80% confidence range of a Monte-Carlo experiment as mentioned above. Then, error propagation to estimate errors on the transport is done in two different ways: first, from the 80% confidence range of a Monte-Carlo experiment repeating 1000 times the transport calculation (Eq. 2), to which we added random noise to the fraction and velocity, within their error limits ($\epsilon _{u_{k,j}}$ and $\epsilon _{f_{k,j}}$); second, from error propagation theory (Supplementary Note 8). Errors displayed in Fig. 5C,D are from the Monte-Carlo propagation which produces larger errors than mathematic propagation theory.

## Supplementary Information

Supplementary Information.

## References

[1] Broecker WS (1991). The great ocean conveyor. Oceanography.

[2] Stommel H (1958). The abyssal circulation. Deep-Sea Res..

[3] Orsi A, Johnson G, Bullister J (1999). Circulation, mixing, and production of Antarctic Bottom Water. Prog. Oceanogr..

[4] Purkey SG, Johnson GC (2013). Antarctic Bottom Water warming and freshening: Contributions to sea level rise, ocean freshwater budgets, and global heat gain. J. Clim..

[5] Desbruyères DG, Purkey SG, McDonagh EL, Johnson GC, King BA (2016). Deep and abyssal ocean warming from 35 years of repeat hydrography. Geophys. Res. Lett..

[6] Patara L, Böning CW (2014). Abyssal ocean warming around Antarctica strengthens the Atlantic overturning circulation. Geophys. Res. Lett..

[7] Stewart AL, Hogg AM (2017). Reshaping the antarctic circumpolar current via Antarctic bottom water export. J. Phys. Oceanogr..

[8] Swingedouw D, Fichefet T, Goosse H, Loutre M-F (2009). Impact of transient freshwater releases in the Southern Ocean on the AMOC and climate. Clim. Dyn..

[9] Meredith MP (2013). Oceanography: Replenishing the abyss. Nat. Geosci..

[10] Carmack EC, Foster TD (1975). Circulation and distribution of oceanographic properties near the Filchner Ice Shelf. Deep Sea Res. Oceanogr. Abstr..

[11] Foster TD, Carmack EC (1976). Frontal zone mixing and Antarctic Bottom Water formation in the southern Weddell Sea. Deep Sea Res. Oceanogr. Abstr..

[12] Weiss R, Östlund H, Craig H (1979). Geochemical studies of the Weddell Sea. Deep Sea Res. Part A Oceanogr. Res. Pap..

[13] Foldvik A, Gammelsrød T, Tørresen T (1985). Circulation and water masses on the southern Weddell Sea shelf. Oceanol. Antarct. Cont. Shelf.

[14] Gordon AL, Huber BA, Hellmer HH, Ffield A (1993). Deep and bottom water of the Weddell Sea’s western rim. Science.

[15] Fahrbach E (1995). Formation and discharge of deep and bottom water in the northwestern Weddell Sea. J. Mar. Res..

[16] Muench RD, Gordon AL (1995). Circulation and transport of water along the western Weddell Sea margin. J. Geophys. Res. Oceans.

[17] Weppernig R, Schlosser P, Khatiwala S, Fairbanks R (1996). Isotope data from Ice Station Weddell: Implications for deep water formation in the Weddell Sea. J. Geophys. Res. Oceans.

[18] Mensch M, Bayer R, Bullister JL, Schlosser P, Weiss RF (1996). The distribution of tritium and CFCs in the Weddell Sea during the mid-1980s. Prog. Oceanogr..

[19] Mensch M, Simon A, Bayer R (1998). Tritium and CFC input functions for the Weddell Sea. J. Geophys. Res. Oceans.

[20] Gordon AL (1998). Interactions at the Antarctic Continental Margin. Western Weddell Sea thermohaline stratification. Ocean, Ice and Atmosphere. Antarct. Res. Ser.

[21] Fahrbach E, Harms S, Rohardt G, Schröder M, Woodgate RA (2001). Flow of bottom water in the northwestern Weddell Sea. J. Geophys. Res. Oceans.

[22] Gordon AL, Visbeck M, Huber B (2001). Export of Weddell Sea deep and bottom water. J. Geophys. Res. Oceans.

[23] Foldvik A, Gammelsrød T, Nygaard E, Østerhus S (2001). Current measurements near Ronne Ice Shelf: Implications for circulation and melting. J. Geophys. Res. Oceans.

[24] Garabato ACN, McDonagh EL, Stevens DP, Heywood KJ, Sanders RJ (2002). On the export of Antarctic Bottom Water from the Weddell Sea. Deep Sea Res. Part II Top. Stud. Oceanogr..

[25] Garabato ACN (2016). The thermodynamic balance of the Weddell Gyre. Geophys. Res. Lett..

[26] Jullion L (2014). The contribution of the Weddell Gyre to the lower limb of the Global Overturning Circulation. J. Geophys. Res. Oceans.

[27] Gill, A. Circulation and bottom water production in the Weddell Sea. In *Deep Sea Research and Oceanographic Abstracts*, vol. 20, 111–140 (Elsevier, 1973).

[28] Foldvik, A. *et al.* Ice shelf water overflow and bottom water formation in the southern Weddell Sea. *J. Geophys. Res. Oceans* **109**, (2004).

[29] Nicholls, K. W. & Østerhus, S. Interannual variability and ventilation timescales in the ocean cavity beneath Filchner-Ronne Ice Shelf, Antarctica. *J. Geophys. Res. Oceans* **109**, (2004).

[30] Huhn O (2008). Evidence of deep-and bottom-water formation in the western Weddell Sea. Deep Sea Res. Part II Top. Stud. Oceanogr..

[31] Meredith MP, Watson AJ, Van Scoy KA, Haine TWN (2001). Chlorofluorocarbon-derived formation rates of the deep and bottom waters of the Weddell Sea. J. Geophys. Res. Oceans.

[32] Akhoudas, C. *et al.* Ice shelf basal melt and influence on dense water outflow in the southern Weddell sea. *J. Geophys. Res. Oceans* **125**, e2019JC015710 (2020).

[33] Purkey SG, Johnson GC (2010). Warming of global abyssal and deep Southern Ocean waters between the 1990s and 2000s: Contributions to global heat and sea level rise budgets. J. Clim..

[34] Rintoul, S. R. Rapid freshening of Antarctic Bottom Water formed in the Indian and Pacific oceans. *Geophys. Res. Lett.* **34**, (2007).

[35] Menezes VV, Macdonald AM, Schatzman C (2017). Accelerated freshening of Antarctic Bottom Water over the last decade in the Southern Indian Ocean. Sci. Adv..

[36] Nicholls, K., Østerhus, S., Makinson, K., Gammelsrød, T. & Fahrbach, E. Ice-ocean processes over the continental shelf of the southern Weddell Sea, Antarctica: A review. *Rev. Geophys.* **47**, (2009).

[37] Georgi DT (1981). Circulation of bottom waters in the southwestern South Atlantic. Deep Sea Res. Part A Oceanogr. Res. Pap..

[38] Nicholls, K., Pudsey, C. & Morris, P. Summertime water masses off the northern Larsen C Ice Shelf, Antarctica. *Geophys. Res. Lett.* **31**, (2004).

[39] Locarnini RA, Whitworth T, Nowlin WD (1993). The importance of the Scotia Sea on the outflow of Weddell Sea Deep Water. J. Mar. Res..

[40] Garabato ACN, Polzin KL, King BA, Heywood KJ, Visbeck M (2004). Widespread intense turbulent mixing in the Southern Ocean. Science.

[41] Heuzé C (2021). Antarctic bottom water and North Atlantic deep water in cmip6 models. Ocean Sci..

[42] Franco BC, Mata MM, Piola AR, Garcia CA (2007). Northwestern Weddell sea deep outflow into the scotia sea during the Austral summers of 2000 and 2001 estimated by inverse methods. Deep Sea Res. Part I Oceanogr. Res. Pap..

[43] Schodlok MP, Hellmer HH, Beckmann A (2002). On the transport, variability and origin of dense water masses crossing the south scotia ridge. Deep Sea Res. Part II Top. Stud. Oceanogr..

[44] Darelius E (2014). On the seasonal signal of the Filchner overflow, Weddell Sea, Antarctica. J. Phys. Oceanogr..

[45] Holland DM, Rosales RR, Stefanica D, Tabak EG (2002). Internal hydraulic jumps and mixing in two-layer flows. J. Fluid Mech..

[46] Ellison T, Turner J (1959). Turbulent entrainment in stratified flows. J. Fluid Mech..

[47] Cenedese C, Whitehead JA, Ascarelli T, Ohiwa M (2004). A dense current flowing down a sloping bottom in a rotating fluid. J. Phys. Oceanogr..

[48] Killworth PD (1977). Mixing of the Weddell Sea continental slope. Deep Sea Res..

[49] Alendal, G., Drange, H. & Haugan, P. M. Modelling of deep-sea gravity currents using an integrated plume model. (1994).

[50] Lago V, England MH (2019). Projected slowdown of Antarctic bottom water formation in response to amplified meltwater contributions. J. Clim..

[51] Moorman R, Morrison AK, McC Hogg A (2020). Thermal responses to Antarctic ice shelf melt in an eddy-rich global ocean-sea ice model. J. Clim..

[52] Munk, W. H. Abyssal recipes. In *Deep Sea Research and Oceanographic Abstracts*, vol. 13, 707–730 (Elsevier, 1966).

[53] Brown PJ (2005). Carbon dynamics of the Weddell Gyre, Southern Ocean. Glob. Biogeochem. Cycl..

[54] Purkey SG, Johnson GC (2012). Global contraction of Antarctic Bottom Water between the 1980s and 2000s. J. Clim..

[55] Heuzé C, Heywood K, Stevens DP, Ridley JK (2013). Southern Ocean bottom water characteristics in CMIP5 models. Geophys. Res. Lett..

[56] Heuzé C, Heywood KJ, Stevens DP, Ridley JK (2015). Changes in global ocean bottom properties and volume transports in CMIP5 models under climate change scenarios. J. Clim..

[57] Sallée J-B (2013). Assessment of Southern Ocean water mass circulation and characteristics in CMIP5 models: Historical bias and forcing response. J. Geophys. Res. Oceans.

[58] De Lavergne C, Palter JB, Galbraith ED, Bernardello R, Marinov I (2014). Cessation of deep convection in the open Southern Ocean under anthropogenic climate change. Nat. Clim. Change.

[59] Meredith MP (1999). Distribution of oxygen isotopes in the water masses of Drake Passage and the South Atlantic. J. Geophys. Res. Oceans.

[60] Rio, m. H., Mulet, S. & Picot, N. New global Mean Dynamic Topography from a GOCE geoid model, altimeter measurements and oceanographic in-situ data. *Proceedings of the ESA Living Planet Symposium, Edinburgh* (2013).

[61] Mackensen A, Hubberten HW, Scheele N, Schlitzer R (1996). Decoupling of $$\delta ^{13}$$C$$_{{\Sigma }{CO}2}$$ and phosphate in recent Weddell Sea deep and bottom water: Implications for glacial Southern Ocean paleoceanography. Paleoceanogr. Paleoclimatol..

[62] Mackensen A (2001). Oxygen and carbon stable isotope tracers of Weddell Sea water masses: New data and some paleoceanographic implications. Deep Sea Res. Part I Oceanogr. Res. Pap..

[63] Meijers, A. J. Jr15006 cruise report. https://www.bodc.ac.uk/resources/inventories/cruise_inventory/reports/jr15006.pdf. Tech. Rep (2016).

[64] Sallée, J.-B. Jr16004 cruise report. https://www.bodc.ac.uk/resources/inventories/cruise_inventory/reports/jr16004.pdf. Tech. Rep (2017).

