# Publisher Correction: Ventilation of the abyss in the Atlantic sector of the Southern Ocean

**DOI:** 10.1038/s41598-021-95949-w

**Published:** 2021-08-12

**Authors:** Camille Hayatte Akhoudas, Jean-Baptiste Sallée, F. Alexander Haumann, Michael P. Meredith, Alberto Naveira Garabato, Gilles Reverdin, Loïc Jullion, Giovanni Aloisi, Marion Benetti, Melanie J. Leng, Carol Arrowsmith

**Affiliations:** 1 CNRS/IRD/MNHN Laboratoire d’Océanographie et du Climat-Expérimentations et Approches Numériques, Sorbonne Université, Paris, France; 2 Atmospheric and Oceanic Sciences Program, Princeton University, Princeton, USA; 3 British Antarctic Survey, Cambridge, UK; 4 School of Ocean and Earth Science, National Oceanography Centre, University of Southampton, Southampton, UK; 5 Institut de Physique du Globe de Paris, Sorbonne Paris Cité, Université Paris Diderot, UMR 7154 CNRS, Paris, France; 6 Institute of Earth Sciences, University of Iceland, Reykjavik, Iceland; 7 NERC Isotope Geosciences Laboratory, British Geological Survey, Nottingham, UK; 8 Centre for Environmental Geochemistry, University of Nottingham, Nottingham, UK

Correction to: *Scientific Reports* 10.1038/s41598-021-86043-2, published online 24 March 2021

The original PDF version of this Article contained errors in Equation 1 where [10*pt*] was incorrectly given.

now reads:

$$\begin{aligned} \left\{ \begin{array}{lll} 1 = f_{DSW}+f_{CDW}+f_{WW}\\[10pt] S_{A}^{obs} = f_{DSW}\cdot S_{A}^{DSW}+f_{CDW}\cdot S_{A}^{CDW}+f_{WW}\cdot S_{A}^{WW}\\[10pt] \delta ^{18}O^{obs} = f_{DSW}\cdot \delta ^{18}O^{DSW}+f_{CDW}\cdot \delta ^{18}O^{CDW}+f_{WW}\cdot \delta ^{18}O^{WW} \end{array} \right. \end{aligned}$$

This has now been corrected in the PDF; the HTML version of the paper was correct from the time of publication.

