# Supplementary material for: Ventilation of the abyss in the Atlantic sector of the Southern Ocean
Source: Sci Rep. 2021 Mar 24;11:6760. doi: 10.1038/s41598-021-86043-2 (PMC7991437; doi:10.1038/s41598-021-86043-2)
Supplement: Supplementary file 1 — Supplementary Information. [file 41598_2021_86043_MOESM1_ESM.pdf]

Supplementary Information :

Ventilation of the abyss in Atlantic sector of the Southern Ocean

Akhoudas et al.

Supplementary Information

Accompanying the article "Ventilation of the abyss in the Atlantic sector of the Southern Ocean" by Akhoudas, C.H., Sallée, J.B., Haumann, F.A., Meredith, P.M., Naveira-Garabato, A., Reverdin, G., Jullion, L., Aloisi, G., Benetti, M.J., Leng, M. and Arrowsmith, C.

In this Supplementary Information section, we present several methods and figures in support of the main article text.

Supplementary Note 1: Note on past studies using CFCs to compute Antarctic Bottom Water production rates in the Weddell Sea

In this study, we compare our results with two important papers that estimated AABW production rates in the Weddell Sea region from CFC data analysis, namely Orsi et al.[1] and Meredith et al.[2]. Those investigations focused on converting a metric ventilation, based on measured CFC concentrations, to a rate of AABW production by making several questionable assumptions on the entrainment ratio.

The assumption of entrainment ratio in Orsi et al.[1] is implicit in the AABW saturation value (i.e. 35%) that they adopted in deriving AABW production rates. While this saturation value is subject to large uncertainties as it depends on a very imperfectly-known water-mass age distribution, it has a very large impact on the resulting AABW transport. The implied entrainment ratio is not directly computed in Orsi et al.[1], but we may estimate it for comparison with our results. Using the DSW saturation level of 50% that Orsi et al.[3] adopted (noting that other studies[4,5] quote somewhat different levels; 55-58%) and a CFC saturation of 0% for CDW, their 35% saturation in AABW converts into an entrainment ratio of 1.43 (1.57-1.66 using the saturation values in Klatt et al.[4] and Mensch et al.[5]). This entrainment ratio is significantly lower than our estimated value of 2.1. Assuming an entrainment ratio of 2.1 is more realistic than 1.43, the AABW saturation level should have been 24% (instead of 35%), which would directly translate into a AABW production rate of 7.1 Sv instead of 4.9 Sv[1], strikingly consistent with our results. Conversely to Orsi et al.[1], Meredith et al.[2] did not assume an AABW saturation value. Instead, they used mass balance across two sections and computed the entrainment ratio based on CFC observations, using a suite of assumptions on the saturation levels of different water-masses. They estimated dilution values on the order of 2.2 at the Crozet-Kerguelen Gap and 2.8 in Vema Channel. Water-mass saturation levels are, again, poorly-known quantities, as they are highly dependent on water-mass age. Meredith et al.[2] finally concluded that the rate of production of newly ventilated AABW is 3.7 $\pm$ 1.6 Sv.

Our study presents a framework to directly compare tracer- and transport-based estimates of ventilation and AABW formation, with no assumptions relating to saturation level or water-mass age. We find that 3.4 $\pm$ 0.6 Sv of AABW is produced locally on the shelves as DSW, and that 3.9 $\pm$ 0.5 Sv is produced by entrainment of CDW, yielding a total for local AABW formation of 7.3 $\pm$ 0.9 Sv in the Weddell Sea. This increases to 8.4 $\pm$ 0.7 Sv for AABW export from the Weddell basin, by additionally considering remote production in the Indo-Pacific sector. These results enable us to revisit previous studies. Thus, the 3.7 $\pm$ 1.6 Sv of DSW production proposed by Meredith et al.[2] is consistent with our estimate of 3.4 $\pm$ 0.6 Sv of newly-ventilated water, i.e. DSW, and that such an estimate is also consistent with the 8.4-9.7 Sv obtained from mass-balance calculations[6–8]. Our results also suggest that the 4.9 Sv of AABW production proposed by Orsi et al.[1] likely assumed an entrainment ratio that is on the low side, which impacts their resulting production rate. Revising the Orsi et al.[1] estimate with an entrainment ratio of 2.1, as found in our study, yields an AABW production rate of 7.4 Sv (or 6.3-6.7 Sv by adopting a DSW

saturation level from other works[4,5]). This is in agreement with our estimate of local Weddell-sourced AABW production of 7.3 ± 0.9 Sv, which is itself consistent with previous estimates from mass-balance calculations.

Supplementary Note 2: Comparison of DSW characteristics from the Larsen continental shelf and Filchner Depression

Multiple lines of evidence obtained downstream of the Larsen continental shelf show DSW formation that contributes to the Weddell-sourced AABW[5,9,10]. In this paper, we assume that the characteristics of DSW acquired in the Filchner Depression sector of the Weddell Sea are a good representation of all DSW of the southern and western shelves of the Weddell Sea. This assumption appears rasonable when gathering the limited DSW characteristics abtained from observations (available from: https://doi.pangaea.de/10.1594/PANGAEA.729699) on the continental slope directly downstream of the Larsen continental shelf[11] (Suppl. Fig. 1A). The Θ–S_A observations (Suppl. Fig. 1B) suggests that their characteristics are consistent with the observations of very cold water formed in interaction with Filchner-Ronne Ice Shelf, within the standard deviation of DSW found in the Filchner Depression.

Supplementary Note 3: Implications of interannual water-mass variability on our assumptions and results

Since the early 1990s to 2014, Antarctic Bottom Water (AABW) has freshened, warmed and declined in volume in the Atlantic sector of the Southern Ocean[12–14]. Abrahamsen et al.[15] indicate that the volume reduction reflects low-frequency variability in the Weddell system, with a recent increase of the AABW supply to the Atlantic Ocean. Here we assess the extent to which such temporal variability may impact our results, which implicitly adopt a steady-state assumption with respect to water-mass sources and production. Temporal variability could affect our results in three ways: (i) our definition of water-mass endmembers that uses invariant characteristics; (ii) our decomposition of the observed water-parcels into the percentage contributions of the different water-mass endmembers; (iii) our transport estimates which are computed for the period 2008 to 2010 rather than the period of the complete observational record.

Issues (i) and (ii) are both linked to our assumption of fixed water-mass endmember characteristics, applied to a decomposition of water-parcels observed over a large time span. To address this issue, we have ascribed conservatively large error bars on our water-mass endmembers properties, so that these error bars encompass the spatio-temporal variability identified over the region and period of our measurements. These error bars are computed from our dataset spanning more than 40 years. To address the possibility that the variability might be larger than that captured by the relatively sparse δ^{18}O dataset, we compare our Θ–S_A dataset. Suppl. Fig. 2A shows the locations of historical Θ and S_A profiles in the region. Θ–S_A diagrams per decade (Suppl. Fig. 2B-F) show that in each decade water-masses as defined in this full historical dataset consistently lie within the error bars of our specified water-mass endmember characteristics. We do not have access to further independent and reliable δ^{18}O observations that we could use to cross-examine our water-mass definition procedure. Furthermore, using this available dataset to produce Θ–S_A diagrams with δ^{18}O in color per decades, Suppl. Fig. 3B-F show that while there is some variability within our specified water-mass endmember characteristics and associated error bars, the decadal variability in δ^{18}O within each water-mass is much lower than the δ^{18}O contrast between water-masses. The standard deviations of δ^{18}O across five decadal means (representative of decadal-scale variability) is 0.02‰ for CDW, 0.06‰ for WW, and 0.03‰ for DSW; much lower than the mean δ^{18}O

contrast between the water masses isotopic composition (i.e. 0.04‰ for CDW, -0.35‰ for WW, and -0.51‰ for DSW; see Methods section). In addition, the standard deviation of δ^{18}O across the five decadal means is encompassed by the δ^{18}O standard deviation used in each water-mass definition (i.e. ± 0.03‰ for CDW, ± 0.07‰ for WW, and ± 0.08‰ for DSW; see Methods section). The slight variability of CDW properties for some decades is indicative of a sampling variability. However, the few observations lying outside of our determined error range of CDW properties (mostly saltier and warmer observations than our definition) remained sparse and the computed error bars cover the majority of this variability. We note nevertheless that bias in our defined CDW properties or on its associated error range caused by uneven sampling, could potentially affect our results on fractions. The sensitivity tests done over the relatively wide error range that we chose for each water-mass however demonstrate that our overall conclusions are robust to such kind of errors on water-mass definition.

Item (iii) directly links to the transport estimates, such that a long-term trend in transport could affect mass-balance diagnostics if a large time span is considered. However, any long-term change in the transport as described earlier[12,13,15] would not affect our estimates, because the inverse calculation is performed using observations spanning only the period 2008 to 2010. Our estimate is therefore not representative of a long-term mean (which would be affected by a long-term trend), but representative of a 2008-2010 mean, and the effects of temporal variability within that period are accounted for in the uncertainty estimates generated by the inversion. Further details on the transport estimates and their errors can be found in the previously published work of Jullion et al.[8].

We are therefore confident that our water-mass definition approach provides a robust representation of the long-term climatological mean state of our study region, and that it is thereby appropriate to decompose observed water-parcel characteristics into endmember fractions.

Supplementary Note 4: Volumetric temperature-salitiny census of the Weddell gyre

We here investigate how much a simple linear combination of three endmembers can explain the full variety of water-masses temperature-salinity characteristics present in the Weddell Sea. To this end we produce a volumetric census of the Weddell gyre in temperature-salinity space using a state of the art ocean climatology (Suppl. Fig. 4A; source data available from: https://www.nodc.noaa.gov/OC5/woa18/woa18data.html). We then superimpose the polygon formed by our three endmembers characteristics and the error bars, which represent the temperature-salinity domain that is mathematically possible to explain from linear combination of our three endmembers (Suppl. Fig. 4B). This polygon covers about 96% of the total volume of the gyre domain. Further, the remaining 4% of waters that lay away from the polygon formed by our three endmembers characteristics and the error bars are mostly in the near-surface layer which is not investigated in our paper.

Supplementary Note 5: Diapycnal mixing deduction in the deep-ocean water-masses

Diapycnal entrainment of CDW into the DSW plumes is expressed in this paper as a volume flux. Assuming that this entrainment occurs evenly over the continental slope, between the 600 m and 3000 m bathymetry contours from the Filchner Depression to the Antarctic Peninsula (Suppl. Fig. 5A), we can estimate a mean diapycnal velocity w, by dividing the volume flux by the total area of 240 x 10^3 km^2, represented as the red area in Suppl. Fig. 5A. On a one-dimensional assumption where we would only consider the vertical dimension, the diapycnal velocity relates the vertical diffusivity, κ, through the simple advection-diffusion

equation:

$$\kappa \cdot \frac{d^2\gamma_n}{dz^2} = w \cdot \frac{d\gamma_n}{dz}. \tag{1}$$

We can therefore further estimate a mean vertical diffusivity associated with our estimated diapycnal entrainment of CDW. We do that by computing the gradient and curvature of a mean neutral density profile (γ_n) over the western continental slope of the Weddell Sea[9] at 28.36 kg m^{-3} γ_n (Suppl. Fig. 5B). κ is then derived from Eq. 1.

Supplementary Note 6: Inter-cruise comparison of δ^{18}O datasets spanning over 40 years

In this paper we use the oxygen isotopic composition δ^{18}O of seawater from a number of different labs around the world. Most of the observations used were however analyzed in only two labs: BGS (British Geological Survey) in the UK, and LOCEAN (Laboratoire d'Océanographie et du Climat Expérimentations et Approches Numeriques) in France. Also, the quantification of transport is done using observations analysed only in these two labs. It is therefore important that we ensure good consistency between measurements done at these two places.

The seawater samples at LOCEAN are analyzed using a PICARRO CRDS instrument (Cavity Ring-Down Spectrometer, model L2130-i Isotopic H_2O) to measure for oxygen isotope composition (δ^{18}O). All seawater samples are analyzed directly with the PICARRO CRDS system using a stainless-steel liner from PICARRO that is inserted in the injection port to avoid salt accumulation in the vaporizer. The use of a liner has the advantage of preserving the accuracy of the seawater isotope analyses as compared to direct injections into the vaporizer. The mesh traps about 80% of the seawater salt[16] and the precision is estimated to be at least 0.06‰. Once the data are calibrated, they are corrected for the analyzer-dependent "sea salt effect" (i.e. δ^{18}O: +0.09‰)[16].

The seawater samples at BGS are analyzed using an IRMS and equilibration method for oxygen isotopes, and do not include analyzer-dependent "sea salt effect" correction. An inter-laboratory comparison between BGS and LOCEAN[16] evaluated the effect of the sea salt on the measurements using equilibration method with IRMS and CRDS techniques. They reveal that the correction associated to measurements carried out with IRMS can differ between labs and estimate that a correction of –0.07‰ for δ^{18}O should be applied for a seawater at salinity $\sim$35 g kg^{-1} for BGS.

During the 2017 WAPITI expedition in the Weddell Sea, samples for water isotopes along the repeated A23 section, located at 30°W across the western Weddell gyre between Orkney Passage and the South Sandwich Trench, were duplicated (Suppl. Fig. 6A) with the objective of inter-laboratory comparison (BGS, UK and LOCEAN, France). The two datasets compare well in the δ^{18}O–S_A space (Suppl. Fig. 6B). We however do observe a mean offset of about 0.09 ± 0.04‰ (Suppl. Fig. 6C, BGS being higher than LOCEAN). The standard deviation of the differences between the two datasets is $\sim$0.04‰, which seems reasonable given the errors associated with both datasets ($\sim$0.04‰ for BGS; $\sim$0.04–0.06‰ for LOCEAN). Given the offset and the comparison study of Benetti et al.[16], we applied an offset of -0.07‰ to the BGS dataset to homogenize the δ^{18}O datasets between LOCEAN and BGS.

In addition to this general offset, we applied further quality control to the BGS datasets. In particular, we identified an offset (Suppl. Fig. 7A,B) between the most recent analysis performed at BGS (post 2016), and the previous ones (2008–2010) run on a different mass spectrometer. When restricting the comparison to overlapping regions (Suppl. Fig. 7C), therefore minimizing the potential influence of regional variability, the offset is still present. Now, looking at this difference in δ^{18}O–γ_n space (Suppl.

Fig. 7D), the offset appears very consistent over a wide range of densities greater than 28 kg m^{-3} γ_n (Suppl. Fig. 7D). Neutral densities greater than 28 kg m^{-3} γ_n encompass water-masses with very different time-scales of ventilation going from tens of years (bottom waters), to hundred of years (CDW). Therefore, if the observed offset in δ^{18}O was physical and due to temporal variability, we would expect that the difference of δ^{18}O between 2008–2010 and 2017 would be different in various density classes. Thus, we also rule out temporal variability as an explanation of the observed δ^{18}O offset between the 2008–2010 and 2017 datasets. We conclude that the observed δ^{18}O offset between the 2008–2010 and 2017 datasets is unphysical (and maybe instrument bias) and needs to be corrected. In order to quantify the correction, we compute the mean offset in the neutral density range 28.15–28.3 kg m^{-3} γ_n (dashed lines in Suppl. Fig. 7E), as it corresponds to the old and stable CDW layer. Based on this comparison, we applied an offset of +0.13‰ to the 2008-2010 datasets to homogenize the δ^{18}O of these two datasets.

Finally, historical datasets from 1973, 1989, 1992 and 1995 are compared with 2017 observations. When considering data in a Θ–S_A space, the observations appear to be consistent despite the span of time (40-20 years) between the cruises and different methods for isotopes analysis (Suppl. Fig. 8A). Suppl. Fig. 8B represents the observations limited in neutral densities greater than 28 kg m^{-3} γ_n where slight variabilities have likely a spatial explanation due to a high mixing behavior of water-masses flowing along the continental slope. Furthermore, in the δ^{18}O–γ_n space all datasets appear to be consistent (Suppl. Fig. 8C). The mean difference for each historical dataset related to 2017 is reported in Suppl. Fig. 8D and then each offset in the neutral density range 28.15–28.3 kg m^{-3} γ_n (as well as dashed lines in Suppl. Fig. 8D), as it corresponds to a water-mass layer that is least variable on timescales of hundreds of years (CDW). Based on the calculation, each offset falls within the instrumental error (i.e for LOCEAN ~0.06‰). We therefore applied no offset correction to these historical datasets, before using them in combination with the other observations.

Supplementary Note 7: Accuracy of tracer characteristics prediction using our source constituents decomposition

In order to evaluate the overall accuracy of our decomposition method, we examine what would be the prediction of the decomposition used in this study in terms of the conservative temperature and dissolved oxygen content. The prediction is then compared to the actual measurements of both tracers. Suppl. Fig. 9 shows histograms of calculated values minus measured ones as well as percentages for dissolved oxygen (Suppl. Fig. 9A-C) and conservative temperature (Suppl. Fig. 9B-D). The comparison is only done for water-masses denser than 28 kg m^{-3} γ_n, which is the focus of this study. Dissolved oxygen prediction is within 15 μmol kg^{-1} (red line as median value on Suppl. Fig. 9A) of the observed value. Given observed dissolved oxygen ranges from ~184 to 328 μmol kg^{-1}, the decomposition allows for a prediction with an accuracy of 10% of this range (red line as median value on Suppl. Fig. 9C). Conservative temperature prediction is within 0.2 °C (red line as median value on Suppl. Fig. 9B) of the observed value. Given observed conservative temperature ranges from ~ -2.3 to 1.93 °C, the decomposition allows for a prediction with an accuracy of 5% this range (red line as median value on Suppl. Fig. 9D).

Supplementary Note 8: Error estimation of the transport calculation

Error estimation of the transport calculation is from the 80% confidence range of a Monte-Carlo experiment repeating 1000 times of the transport calculation in the main text. In addition, here we examine the error propagated mathematically. From error propagation theory, one can write the covariance of transport T across the ANDREX/I06S section in terms of covariance

of $f_{k,j}$ the fraction of the "source" water mass estimated at station j, level k and $u_{k,j}$ the corresponding adjusted geostrophic velocity from Jullion et al.[8] ($\mathscr{A}_{k,j}$, the area defined by vertical spacing and station spacing, has no associated error):

$$cov(T) = J\ cov(C)\ J^T \tag{2}$$

with:

$$J = \begin{pmatrix} \frac{\partial T}{\partial f_{k,j}} \\ \vdots \\ \vdots \\ \frac{\partial T}{\partial u_{k,j}} \\ \vdots \\ \vdots \end{pmatrix}$$

$$cov(C) = \left[\begin{array}{c|c} cov(f_{k,j}) & 0 \\ \hline 0 & cov(u_{k,j}) \end{array}\right]$$

Assuming that errors in fractions and velocity have no spatial correlation, one can write: $cov(f_{k,j}) = Diag(\varepsilon^2_{f_{k,j}})$, and $cov(u_{k,j}) = Diag(\varepsilon^2_{u_{k,j}})$, where $Diag$ is a diagonal matrix.

By combining these terms in Eq. 2, we find that the error on the Transport T (total and for each water-mass endmember, Suppl. Fig. 10), expressed as its standard deviation, std(T), is:

$$std(T) = \sqrt{cov(T)}, \tag{3}$$

with:

$$cov(T) = \sum_k^n \sum_j^m \left[\varepsilon_{f_{k,j}} \times u_{k,j} \times \mathscr{A}_{k,j}\right]^2 + \left[f_{k,j} \times \varepsilon_{u_{k,j}} \times \mathscr{A}_{k,j}\right]^2 \tag{4}$$

References

1. Orsi, A., Johnson, G. & Bullister, J. Circulation, mixing, and production of Antarctic Bottom Water. *Prog. Oceanogr.* **43**, 55–109 (1999).

2. Meredith, M. P., Watson, A. J., Van Scoy, K. A. & Haine, T. W. N. Chlorofluorocarbon-derived formation rates of the deep and bottom waters of the Weddell Sea. *J. Geophys. Res. Ocean.* **106**, 2899–2919 (2001).

3. Orsi, A. H., Smethie Jr, W. M. & Bullister, J. L. On the total input of Antarctic waters to the deep ocean: A preliminary estimate from chlorofluorocarbon measurements. *J. Geophys. Res. Ocean.* **107**, 31–1 (2002).

4. Klatt, O. *et al.* Repeated CFC sections at the Greenwich Meridian in the Weddell Sea. *J. Geophys. Res. Ocean.* **107**, 5–1 (2002).

5. Mensch, M., Simon, A. & Bayer, R. Tritium and CFC input functions for the Weddell Sea. *J. Geophys. Res. Ocean.* **103**, 15923–15937 (1998).

6. Garabato, A. C. N. *et al.* The thermodynamic balance of the Weddell Gyre. *Geophys. Res. Lett.* **43**, 317–325 (2016).

7. Garabato, A. C. N., McDonagh, E. L., Stevens, D. P., Heywood, K. J. & Sanders, R. J. On the export of Antarctic bottom water from the Weddell Sea. *Deep. Sea Res. Part II: Top. Stud. Oceanogr.* **49**, 4715–4742 (2002).

8. Jullion, L. *et al.* The contribution of the Weddell Gyre to the lower limb of the Global Overturning Circulation. *J. Geophys. Res. Ocean.* **119**, 3357–3377 (2014).

9. Weppernig, R., Schlosser, P., Khatiwala, S. & Fairbanks, R. Isotope data from Ice Station Weddell: Implications for deep water formation in the Weddell Sea. *J. Geophys. Res. Ocean.* **101**, 25723–25739 (1996).

10. Huhn, O. *et al.* Evidence of deep-and bottom-water formation in the western Weddell Sea. *Deep. Sea Res. Part II: Top. Stud. Oceanogr.* **55**, 1098–1116 (2008).

11. Absy, J. M., Schröder, M., Muench, R. D. & Hellmer, H. H. Physical oceanography from 120 CTDs, 79 water bottles stations, and 30 helicopter-borne CTDs during POLARSTERN cruise ANT-XXII/2 (ISPOL) (2008).

12. Johnson, G. C., McTaggart, K. E. & Wanninkhof, R. Antarctic Bottom Water temperature changes in the western South Atlantic from 1989 to 2014. *J. Geophys. Res. Ocean.* **119**, 8567–8577 (2014).

13. Purkey, S. G. & Johnson, G. C. Global contraction of Antarctic Bottom Water between the 1980s and 2000s. *J. Clim.* **25**, 5830–5844 (2012).

14. Johnson, G. C., Purkey, S. G. & Toole, J. M. Reduced antarctic meridional overturning circulation reaches the north atlantic ocean. *Geophys. Res. Lett.* **35** (2008).

15. Abrahamsen, E. P. *et al.* Stabilization of dense Antarctic water supply to the Atlantic Ocean overturning circulation. *Nat. Clim. Chang.* **9**, 742–746 (2019).

16. Benetti, M. *et al.* Inter-comparison of salt effect correction for δ^{18}o and δ^{2}h measurements in seawater by crds and irms using the gas-h^{2}o equilibration method. *Mar. Chem.* **194**, 114–123 (2017).

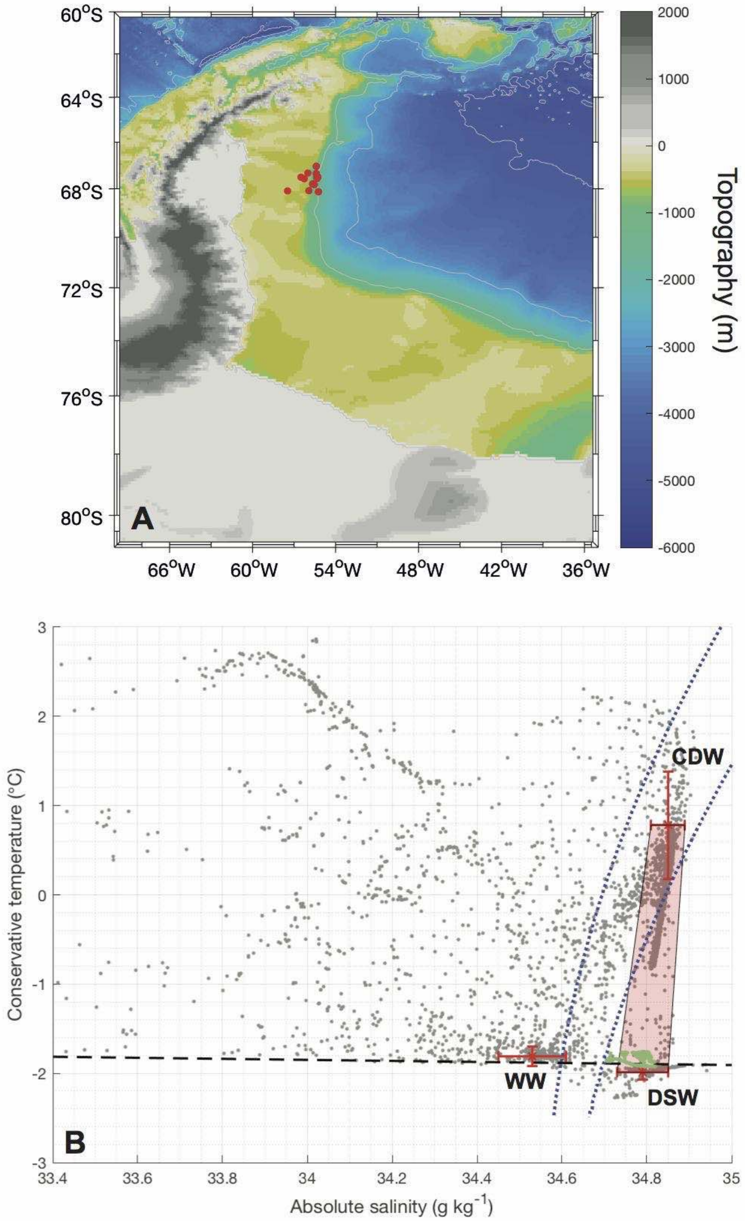

Supplementary Figure 1. (A) Topography in the southeastern sector of the Weddell Sea. The red dots show observations obtained on the continental slope directly downstream of the Larsen continental shelf[11]. (B) Θ–S_A diagram showing observation from the complete dataset used in the article shown in figure 1B. Neutral density surfaces 28 and 28.27 kg m^{-3} γ_n are superimposed as blue dashed curves and the surface freezing line as black dashed line. Mean and standard deviation of "pure" waters-masses (CDW, DSW and WW) characteristics are indicated as red crosses. The green dots represent the Θ–S_A characteristics of DSW in the Larsen region. Map was produced using the software Matlab R2019a.

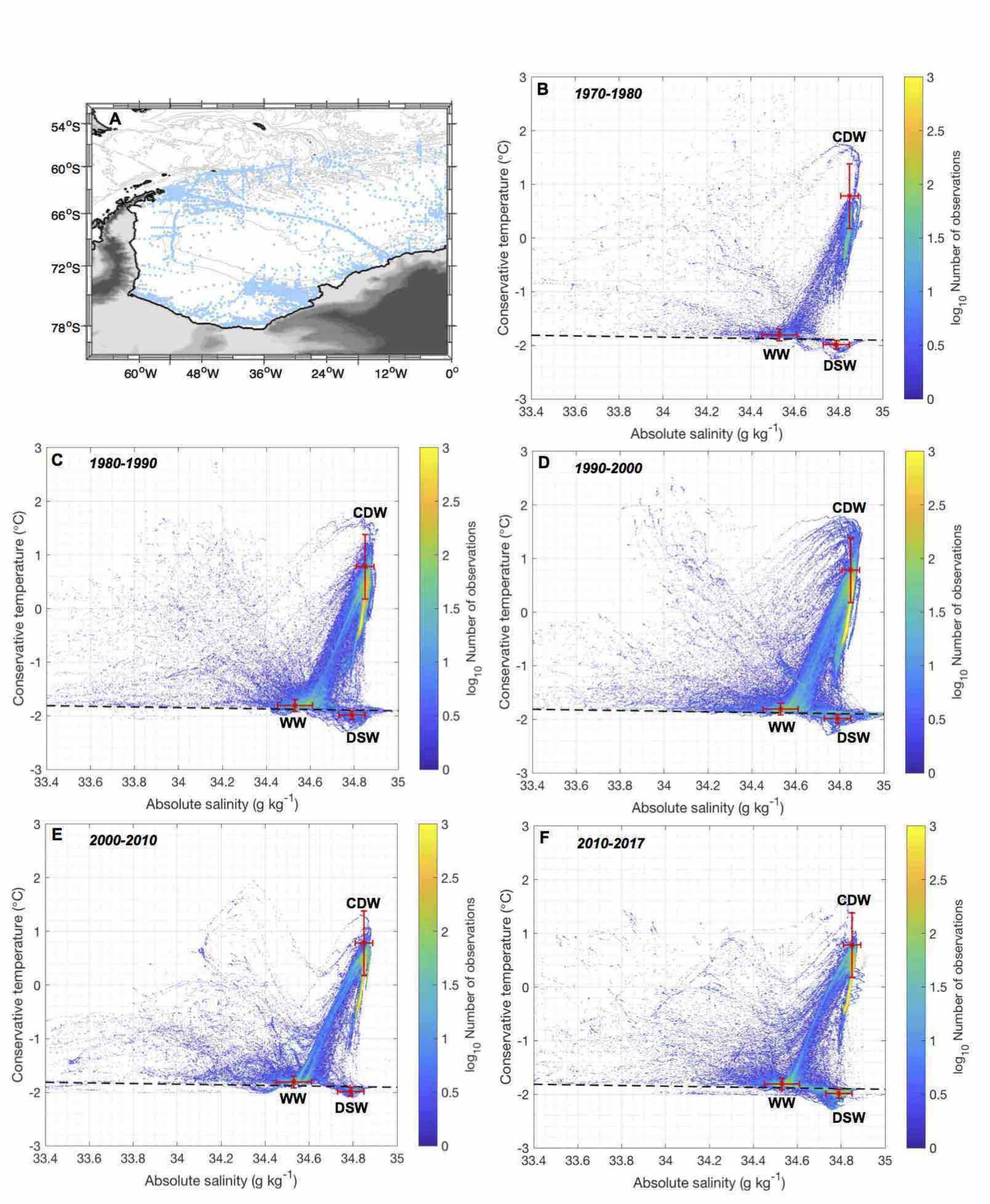

Supplementary Figure 2. (A) The Weddell and Scotia Seas sector with blue dots showing the ship-based observations in the western Weddell gyre. Log10 of the observations' number is represented in color for each decade in Θ–S_A diagrams (B from 1970 to 1980, C from 1980 to 1990, D from 1990 to 2000, E from 2000 to 2010 and F from 2010 to 2017). Mean and standard deviation of source water-masses (CDW, DSW and WW) characteristics are indicated as red crosses. Map was produced using the software Matlab R2019a.

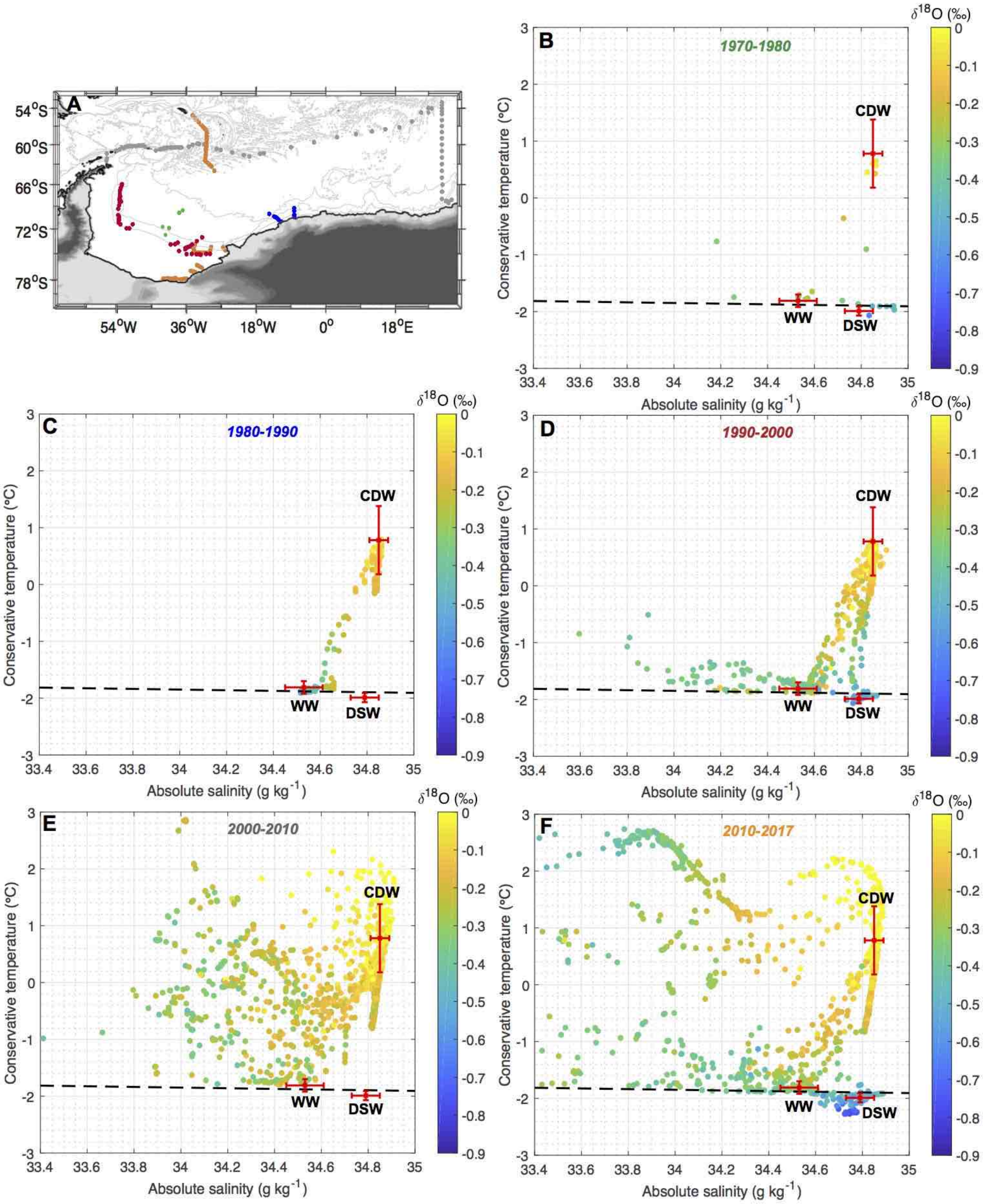

Supplementary Figure 3. The Weddell and Scotia Seas sector with dots showing the position of the compiled observation database used in this study, with color code corresponding to different decades: (IWSOE 73 observations in green) 1970-1980, (SR02 observations in blue) 1980-1990, (Ice Station Weddell and SR02 observations in red) 1990-2000, (ANDREX/I06S observations in gray) 2000-2010 and (A23 and WAPITI observations in orange) 2010-2017. δ^{18}O is represented in color for each decade in Θ–S_A diagrams (B from 1970 to 1980, C from 1980 to 1990, D from 1990 to 2000, E from 2000 to 2010 and F from 2010 to 2017). Mean and standard deviation of source water-masses (CDW, DSW and WW) characteristics are indicated as red crosses. Map was produced using the software Matlab R2019a.

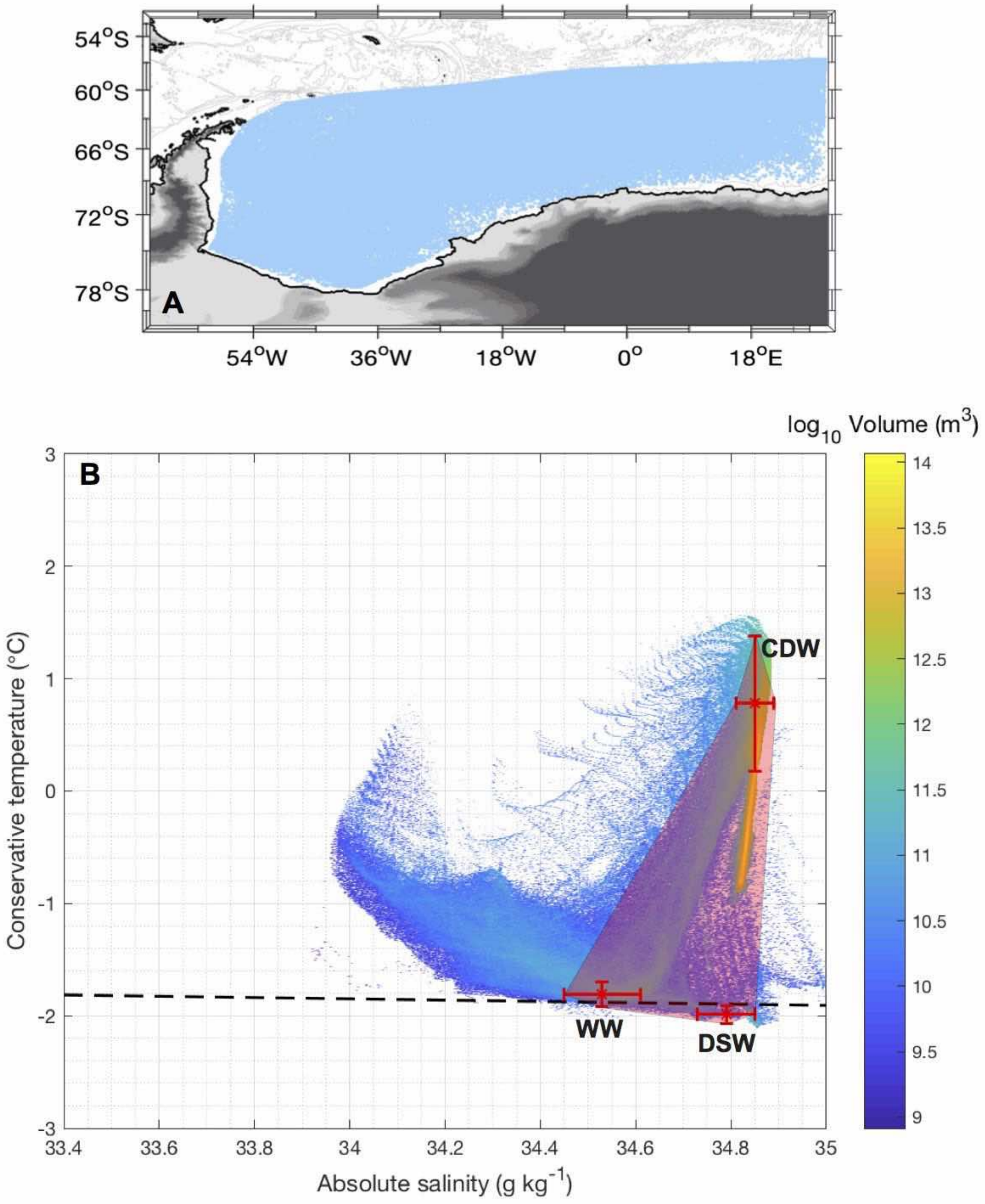

Supplementary Figure 4. (A) The Weddell and Scotia Seas sector with the blue dots showing the observations for the Weddell gyre from *in situ* profile data. The observations correspond to climatological mean temperature and salinity averaged over the 1981-2010 period (1/4° grid) for the gyre domain. Log10 of gyre observations' volume (1.2 x 10^{17} m^3) is represented in a Θ–S_A diagram in panel B and mean and standard deviation of "source" water-masses (CDW, DSW and WW) characteristics are indicated as red crosses. The "source" water-masses triangle that we consider in the mass balance calculation is defined as the red patch and represent a volume of 1.1 x 10^{17} m^3. Map was produced using the software Matlab R2019a.

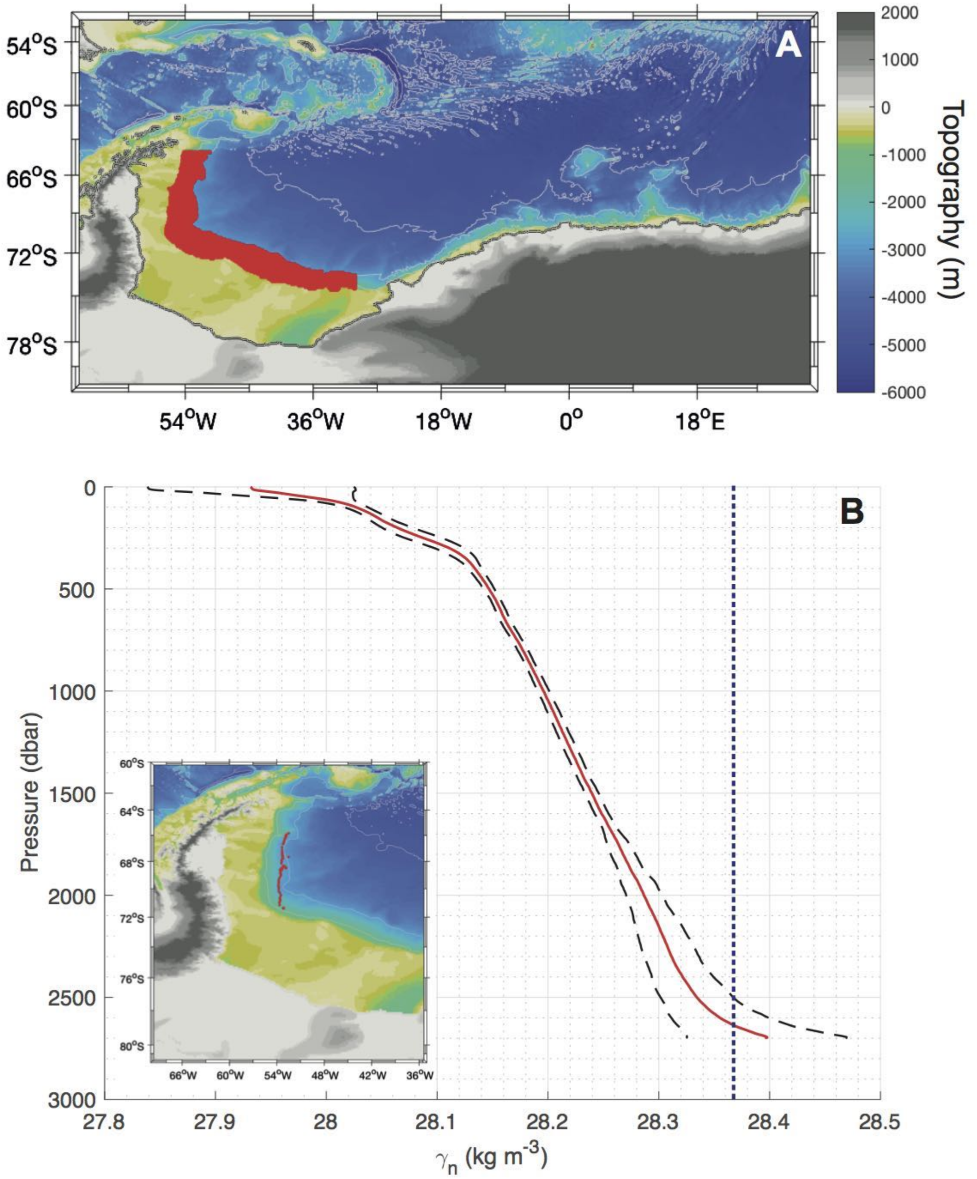

Supplementary Figure 5. (A) Topography in the Atlantic sector of the Southern Ocean with the continental slope between the 600 m and 3000 m bathymetry contours covering an area of about 240 10^3 km^2 considered as red patch. (B) Ensemble-mean (red curve) and standard deviation (black dashed curves) vertical profiles of neutral density γ_n in the continental slope of the western Weddell Sea (red dots on the corner map). Neutral density surface 28.36 kg m^{-3} γ_n is shown as blue dashed line. Maps were produced using the software Matlab R2019a.

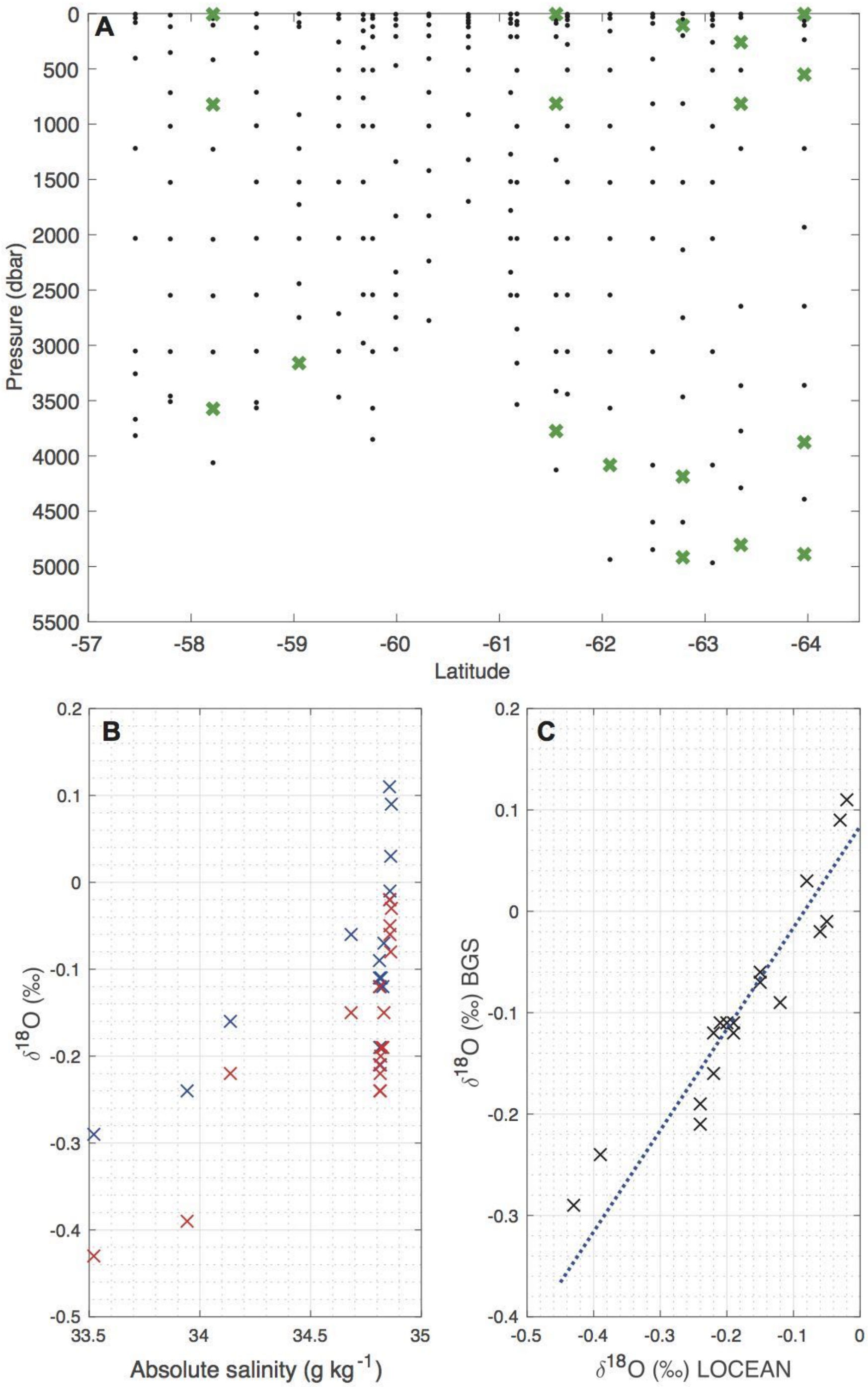

Supplementary Figure 6. (A) Location and depth of duplicated samples (green crosses) considered in the inter-comparison work along the A23 section in 2017 at 30°W between the Orkney Passage and the South Sandwich Trench. (B) Comparison of measurements from the BGS (blue crosses) and LOCEAN (red crosses) laboratories at the same location and depth in a δ^{18}O–S_A space. (C) The mean offset of 0.09‰ shown by the blue dashed line.

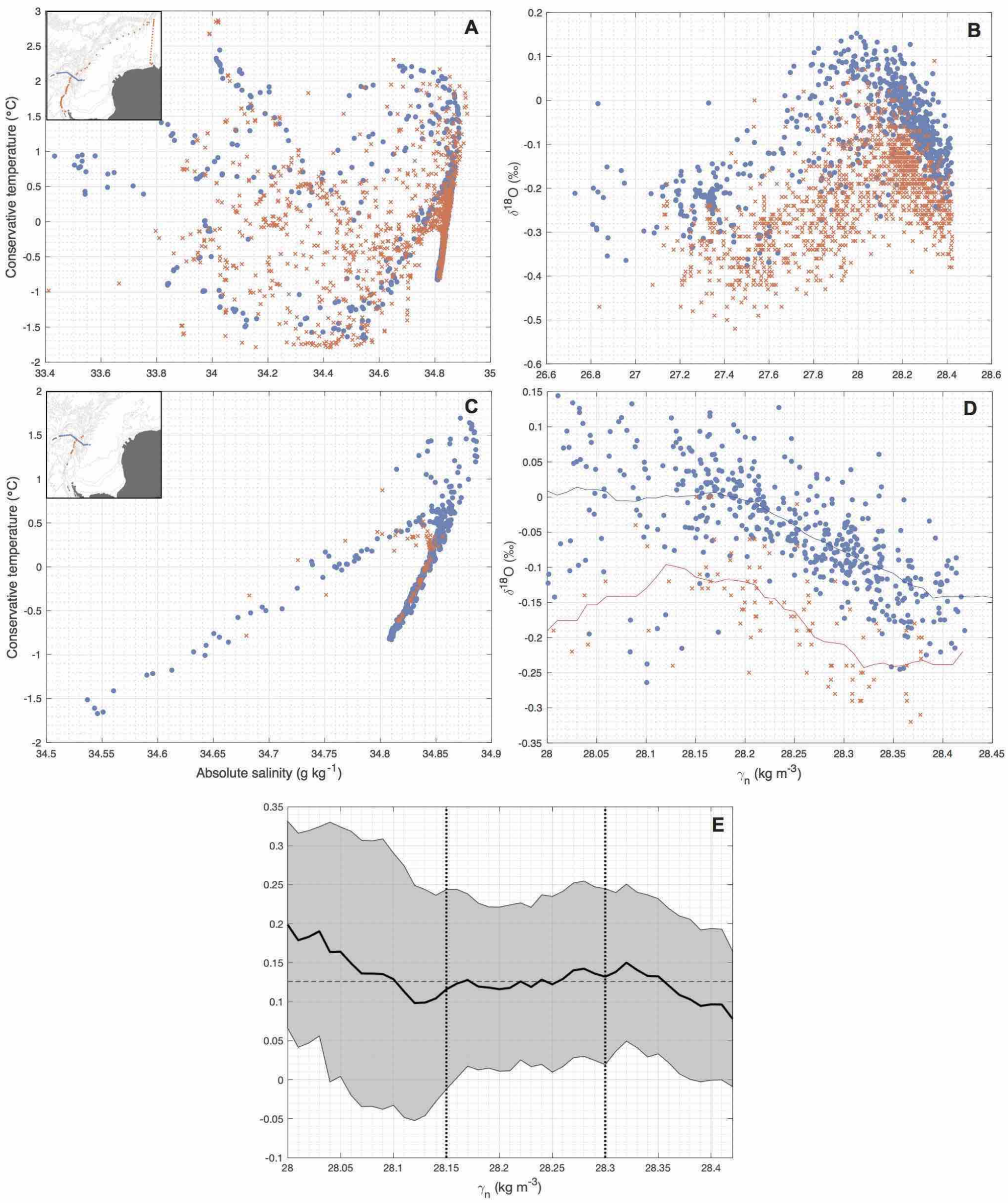

Supplementary Figure 7. Θ–S_A (A) and δ^{18}O–γ_n (B) characteristics of all observations sampled in the Weddell gyre in 2008, 2009 and 2010 (orange crosses) and 2016/2017 (blue dots). Θ–S_A (C) and δ^{18}O–γ_n (D) characteristics of observations with γ_n > 28 kg m^{-3} sampled in the Weddell gyre in 2010 but limited to the longitude range between 40°W and 25°W (orange crosses) and 2016/2017 (blue dots). On panel (E) the mean δ^{18}O value is computed per 0.01 γ_n range and plotted as plain lines. The black line in panel (E) is the mean offsets per density range, along with the associated standard deviation (gray shading). Neutral density surface 28.15 and 28.30 kg m^{-3} γ_n are superimposed as black dashed lines as boundaries of CDW layer.

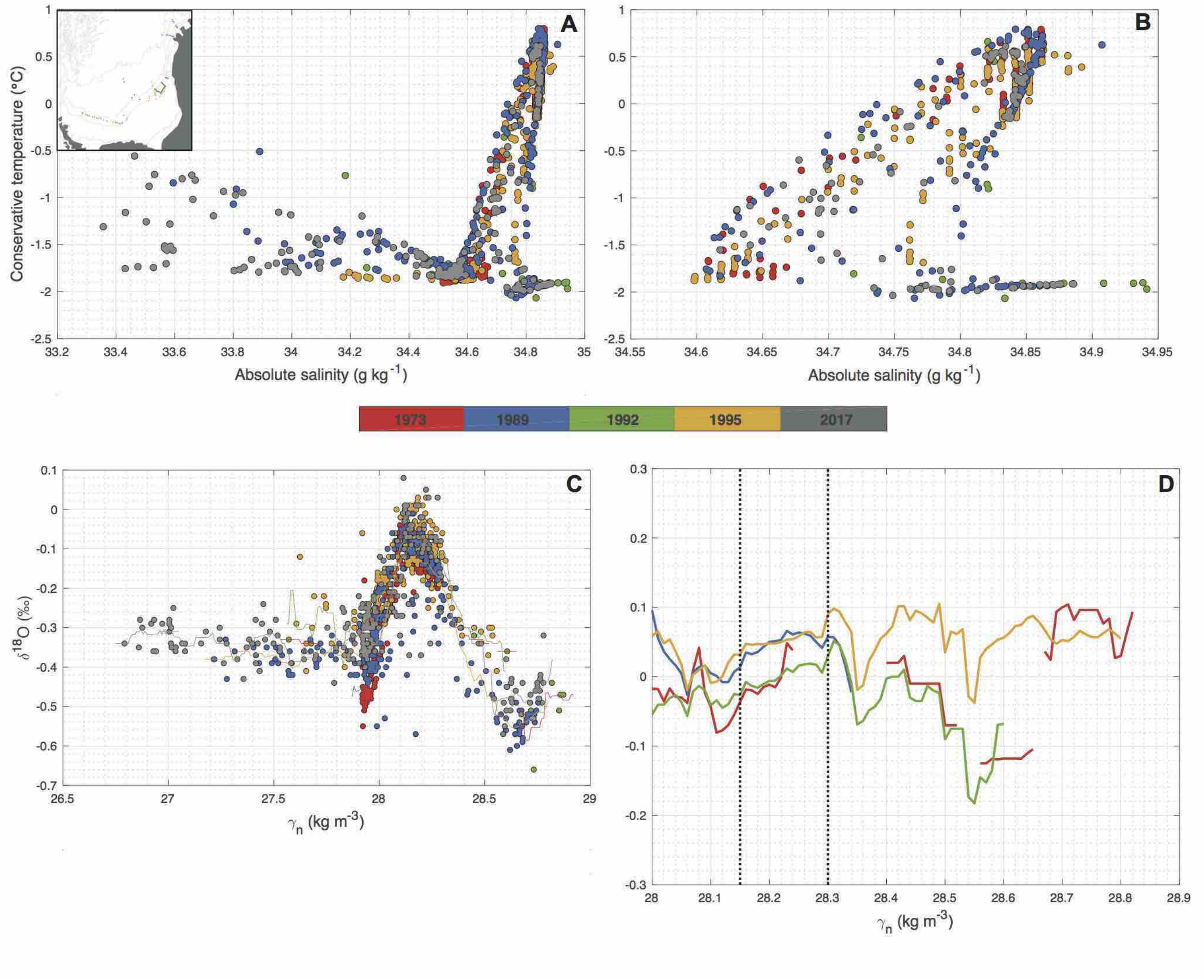

Supplementary Figure 8. Θ–S_A characteristics of all observations (A) and of observations with γ_n > 28 kg m^{-3} (B) sampled in the southeastern Weddell Sea in 1973 (red dots), 1989 (blue dots), 1992 (green dots), 1995 (orange dots) and 2017 (gray dots). δ^{18}O–γ_n characteristics of all observations (C) with the mean δ^{18}O values computed per 0.01 γ_n range and plotted as plain lines. The colored lines in panel (D) are the mean offsets per density range. Red line: 2017 minus 1973 / blue line: 2017 minus 1989 / green line: 2017 minus 1992 / orange line: 2017 minus 1995. Neutral density surface 28.15 and 28.30 kg m^{-3} γ_n are superimposed as black dashed lines as boundaries of CDW layer.

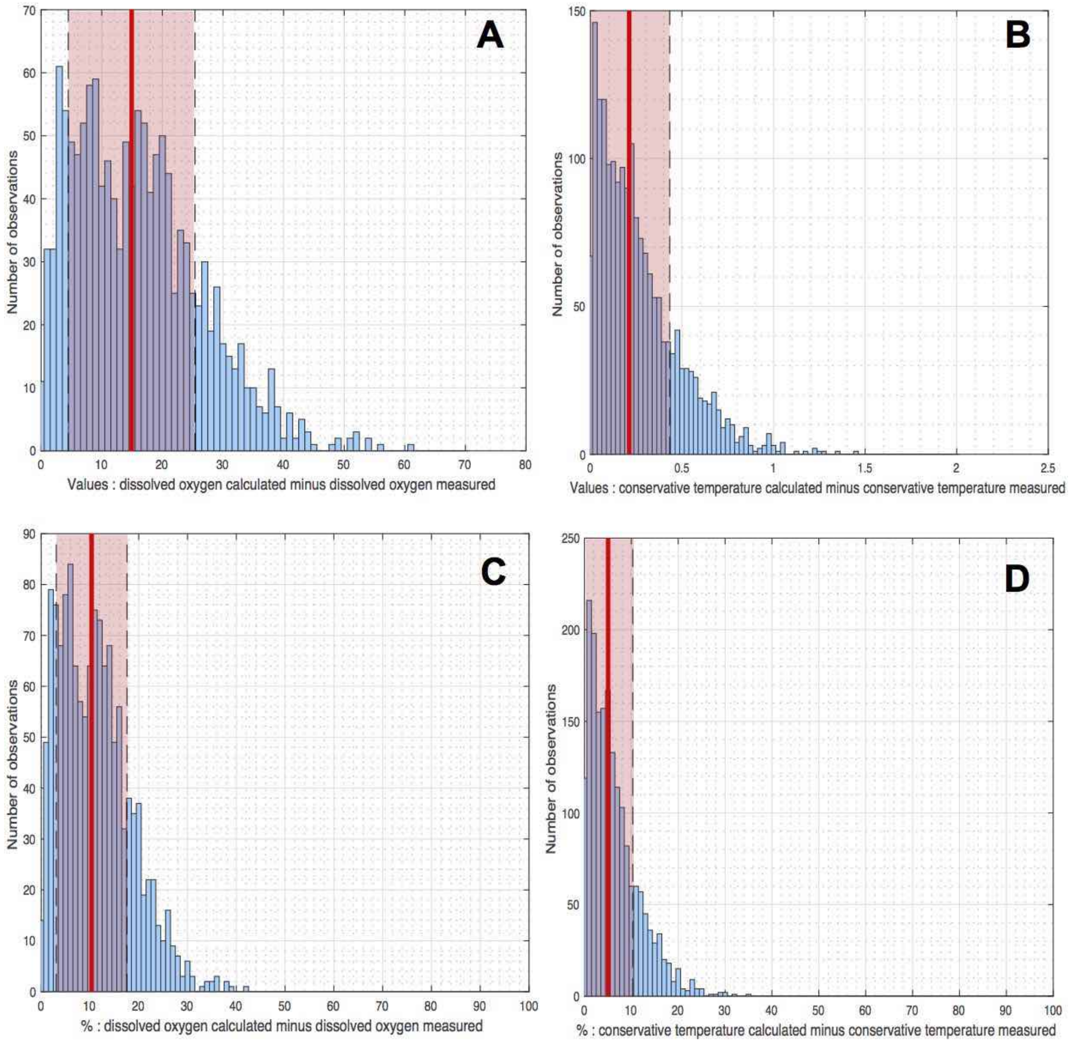

Supplementary Figure 9. Histograms of calculated values minus measured values for dissolved oxygen (A) and conservative temperature (B). Percentages of calculated values minus measured values for dissolved oxygen (C) and conservative temperature (D). The comparison is only done for water-masses denser than 28 kg m^{-3} γ_n. For each panel, the red line and red shading represent the medians and standard deviations respectively.

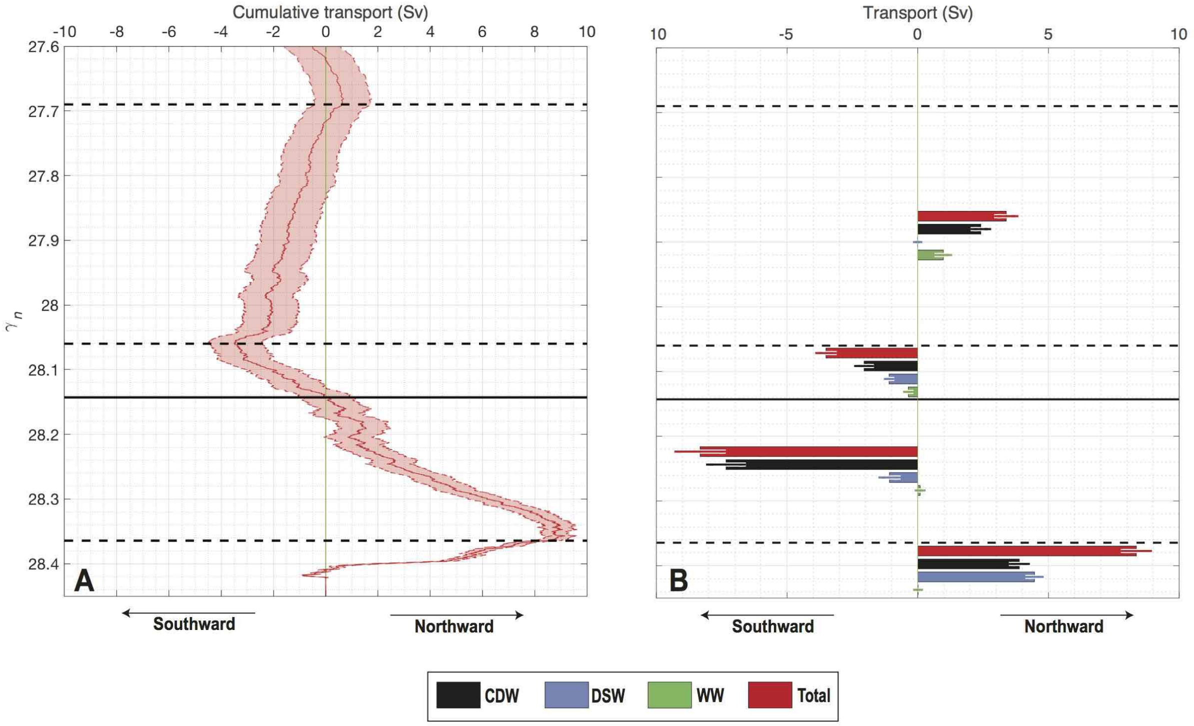

Supplementary Figure 10. (A) Total cumulative transport from the bottom ocean upward, summed across the ANDREX/I06S section, computed in neutral density coordinates. Shading indicates errors propagated mathematically. Positive (negative) transport are directed out of (into) the gyre. (B) Net transports across the ANDREX/I06S into four density layers: 27.69-28.06 kg m^{-3} γ_n; 28.06-28.14 kg m^{-3} γ_n; 28.14-28.36 kg m^{-3} γ_n; and denser than 28.36 kg m^{-3} γ_n. For each of these layers, the total net transport (red bars) are decomposed into their end-member contributions: (black) CDW, (blue) DSW, (green) WW. Positive (negative) transport are directed out of (into) the gyre.
